# Personalized Medicine in Mitochondrial Health and Disease: Molecular Basis of Therapeutic Approaches Based on Nutritional Supplements and Their Analogs

**DOI:** 10.3390/molecules27113494

**Published:** 2022-05-29

**Authors:** Vincenzo Tragni, Guido Primiano, Albina Tummolo, Lucas Cafferati Beltrame, Gianluigi La Piana, Maria Noemi Sgobba, Maria Maddalena Cavalluzzi, Giulia Paterno, Ruggiero Gorgoglione, Mariateresa Volpicella, Lorenzo Guerra, Domenico Marzulli, Serenella Servidei, Anna De Grassi, Giuseppe Petrosillo, Giovanni Lentini, Ciro Leonardo Pierri

**Affiliations:** 1 Department of Biosciences, Biotechnologies, Biopharmaceutics, University of Bari Aldo Moro, Via E. Orabona, 4, 70125 Bari, Italy; v.tragni@ibiom.cnr.it (V.T.); lucas.cafferatibeltrame@uniba.it (L.C.B.); gianluigi.lapiana@uniba.it (G.L.P.); maria.sgobba@uniba.it (M.N.S.); rugorgo@hotmail.it (R.G.); mariateresa.volpicella@uniba.it (M.V.); lorenzo.guerra1@uniba.it (L.G.); anna.degrassi@uniba.it (A.D.G.); 2 Institute of Biomembranes, Bioenergetics and Molecular Biotechnologies (IBIOM), National Research Council (CNR), 70126 Bari, Italy; d.marzulli@ibiom.cnr.it; 3 Fondazione Policlinico Universitario A. Gemelli IRCCS, 00168 Rome, Italy; guidoalessandro.primiano@policlinicogemelli.it (G.P.); serenella.servidei@policlinicogemelli.it (S.S.); 4 Dipartimento Universitario di Neuroscienze, Università Cattolica del Sacro Cuore, 00168 Rome, Italy; 5 Department of Metabolic Diseases, Clinical Genetics and Diabetology, Giovanni XXIII Children Hospital, Azienda Ospedaliero-Universitaria Consorziale, Via Amendola 207, 70126 Bari, Italy; albina.tummolo@policlinico.ba.it (A.T.); paternogiulia@icloud.com (G.P.); 6 Department of Pharmacy—Pharmaceutical Sciences, University of Bari Aldo Moro, Via E. Orabona 4, 70125 Bari, Italy; mariamaddalena.cavalluzzi@uniba.it

**Keywords:** mitochondrial impairment, mitochondrial dysfunction, mitochondrial diseases, vitamins, cofactors, dietary supplement, aminoacyl tRNA synthetase, phospholipids, peptide-based treatments, CRAT deficiency, SLC25A10 and DIC deficiency, MERRF, MELAS, Leigh syndrome, Leigh-like syndromes, MEGDEL, encephalomyopathies, antioxidant cocktails, mitochondrial carriers, complex I, type I NADH dehydrogenase

## Abstract

Mitochondrial diseases (MDs) may result from mutations affecting nuclear or mitochondrial genes, encoding mitochondrial proteins, or non-protein-coding mitochondrial RNA. Despite the great variability of affected genes, in the most severe cases, a neuromuscular and neurodegenerative phenotype is observed, and no specific therapy exists for a complete recovery from the disease. The most used treatments are symptomatic and based on the administration of antioxidant cocktails combined with antiepileptic/antipsychotic drugs and supportive therapy for multiorgan involvement. Nevertheless, the real utility of antioxidant cocktail treatments for patients affected by MDs still needs to be scientifically demonstrated. Unfortunately, clinical trials for antioxidant therapies using α-tocopherol, ascorbate, glutathione, riboflavin, niacin, acetyl-carnitine and coenzyme Q have met a limited success. Indeed, it would be expected that the employed antioxidants can only be effective if they are able to target the specific mechanism, i.e., involving the central and peripheral nervous system, responsible for the clinical manifestations of the disease. Noteworthily, very often the phenotypes characterizing MD patients are associated with mutations in proteins whose function does not depend on specific cofactors. Conversely, the administration of the antioxidant cocktails might determine the suppression of endogenous oxidants resulting in deleterious effects on cell viability and/or toxicity for patients. In order to avoid toxicity effects and before administering the antioxidant therapy, it might be useful to ascertain the blood serum levels of antioxidants and cofactors to be administered in MD patients. It would be also worthwhile to check the localization of mutations affecting proteins whose function should depend (less or more directly) on the cofactors to be administered, for estimating the real need and predicting the success of the proposed cofactor/antioxidant-based therapy.

**Contents**


1. Introduction·············································································································································································································
2

2. Different Molecular Mechanisms Associated with the Same Phenotype·········································································································
2

3. Symptomatic Treatments Based on Antioxidant Cocktails································································································································
3
4. Small Molecules, Vitamins and Cofactors Administered as Single Molecules or as a Cocktail to Patients Affected by MD····················
3

 4.1. Vitamin B1—Thiamine····················································································································································································
17
 4.2. Vitamin B2—Riboflavin··················································································································································································
19

 4.3. Vitamin B3—Niacin························································································································································································
20

 4.4. Vitamin B5—Pantothenic Acid······································································································································································
21

 4.5. Vitamin B6—Pyridoxine················································································································································································
21

 4.6. Vitamin B7—Biotin·························································································································································································
22

 4.7. Vitamin B9—Folic Acid··················································································································································································
22

 4.8. Vitamin B12—Cobalamin···············································································································································································
22

 4.9. Vitamin C—L-Ascorbic Acid·········································································································································································
23

 4.10. Lipoic Acid····································································································································································································
23

 4.11. Vitamin A—Retinol······················································································································································································
24

 4.12. Vitamin D—Calciferol·················································································································································································
24

 4.13. Vitamin E—α-Tocopherol···········································································································································································
24

 4.14. Vitamin K—Phylloquinone··········································································································································································
25

 4.15. Coenzyme Q10 (Reduced, as Ubiquinol; Oxidized, as Ubiquinone)······································································································
25

 4.16. L-Carnitine/Acetyl-L-Carnitine··································································································································································
26

5. Molecular Mechanisms of ROS Production and Mitochondrial Damage Observed in Cells from Mitochondrial Patient Tissues·········
27

6. The Need for a Structural Analysis for Evaluating the Real Need for Vitamin Supplementation·····························································
27

7. Missense Mutations Responsible for Severe Encephalomyopathies: The Case of *MT-ND5*, *NDUFAF6* and *SERAC1*·····························
29

8. The Case of Carnitine O-Acetyltransferase (CRAT) Deficiency·····················································································································
31

9. SLC25A10 Dicarboxylate Carrier (DIC) Deficiency··········································································································································
32

10. MDs Depending on AA-tRNA Mutations or AA-tRNA Synthetase Mutations·························································································
32

11. CoQ Analogs and Mitochondrial Delivery Systems: Organic/Inorganic Chemicals for Stimulating Mitochondrial Activity··············
37

12. Conclusions·········································································································································································································
38

Abbreviations············································································································································································································
39

References··················································································································································································································
40


## 1. Introduction

Mitochondria are organelles known for being the powerhouse of the cell, and they are present in almost all human cells except for erythrocytes. The mitochondrial oxidative phosphorylation (OXPHOS) process plays a pivotal role in cellular energy production by coupling the respiratory chain electron transfer to oxygen with the production of ATP. These organelles are also involved in various cellular metabolic pathways and play a critical role in apoptosis in phylogenetically distant eukaryotic organisms [1,2,3,4,5]. Thus, mitochondrial dysfunction may adversely affect cell physiology, and the abilities of mitochondria to provide the cell with the proper amount of ATP and to make correct life-or-death decisions are vital for supporting a healthy life. Mitochondrial diseases (MDs) are, in the vast majority, clinically devastating human disorders that can occur at any age with a wide range of clinical symptoms that generally involve tissues highly dependent on aerobic metabolism.

## 2. Different Molecular Mechanisms Associated with the Same Phenotype

MDs are long-term, genetic disorders characterized by alterations in mitochondrial function that may result from mutations affecting nuclear or mitochondrial genes, encoding mitochondrial proteins or non-protein-coding RNA [6,7].

Different clinical manifestations of severe encephalomyopathies characterize Leigh or Leigh-like syndromes (https://www.omim.org/entry/256000; accessed on 8 April 2022), the commonest syndromic presentations of pediatric MD, mitochondrial encephalopathy, lactic acidosis, stroke-like episodes (MELAS, https://www.omim.org/entry/540000, accessed on 8 April 2022) and myoclonic epilepsy with ragged-red fibers (MERRF; https://www.omim.org/entry/545000 accessed on 8 April 2022) syndromes, more commonly associated with late-onset pattern [8,9,10,11,12,13,14,15,16,17,18,19].

At the molecular level, Leigh syndrome can be caused by numerous mutations, both in nuclear and mitochondrial genes. Mutations involving complex I subunits or mutations affecting other proteins localized within mitochondria were held responsible for Leigh [9,13,14,15,20] or a Leigh-like syndrome with severe encephalomyopathy [20,21,22,23]. Several epileptic encephalopathies share phenotypical traits with the Leigh syndrome, with mutations affecting both coding and non-coding mitochondrial or nuclear DNA [12,19,21,24,25,26]. 

MELAS and MERRF syndromes are instead caused by mutations in mitochondrial DNA coding for tRNAs. In particular, MELAS syndrome is associated in more than 80% of cases with the m.3243A>G (pathogenic variant within MT-TL1, encoding mt-tRNALeu(UUR)) with the consequent impairment of the folding of different respiratory chain complexes [25]. Conversely, MERRF syndrome is commonly related to m.8344A>G mutation (within MT-TK, encoding mt-tRNALys) [25] and a predominant involvement of respiratory chain complex IV [13,18,19,25,27].

## 3. Symptomatic Treatments Based on Antioxidant Cocktails

Even if new molecular therapeutic strategies are available [28,29] and recent developments in the reproductive options for patients with mitochondrial myopathies provide a possibility for preventing the transmission of the mutation to the next generation [30], there are currently no effective treatments approved and available for the majority of patients with MDs. In fact, the currently employed therapeutic options focus on the symptomatic management of disease manifestations, aiming to improve the patients’ quality of life.

Regular aerobic exercise, when possible, is suggested and was shown to enhance exercise tolerance and improve the fatigue MD common condition [31,32]. Similarly, surgical interventions for eyelid ptosis, another common manifestation in patients affected by MDs [33], can significantly improve patients’ quality of life. 

From a purely pharmacological point of view, while few and selected drugs (e.g., valproic acid) should be avoided or used with caution in this group of neurogenetic diseases [34,35], most MD patients are treated with antioxidant cocktails (mito-cocktails) or the related dietary supplements, only based on the concept of a primary defect in oxidative phosphorylation (OXPHOS), a common pathogenic condition for all MDs.

Nevertheless, the real usefulness of treatments based on the administration of antioxidant cocktails (consisting of a-tocopherol, ascorbate, glutathione, riboflavin, niacin, vitamin E, acetyl-carnitine and coenzyme Q) to patients affected by MDs still needs to be scientifically and clinically demonstrated [36,37,38]. In general, antioxidant cocktails are frequently administered in combination with symptomatic therapy based on antiepileptic drugs and other chemicals aiming at alleviating the systemic manifestations of these diseases [37,38,39].

The explanation of the limited success of these therapeutic approaches could be related to the fact that the employed antioxidants can only be effective if the specific mechanism causing the clinical manifestations depends on the failed interactions between the administered cofactor/vitamin and its binding site in the mutated protein or on the scavenging abilities of the employed small molecules to reduce the reactive species produced by the mutated protein. Thus, it might be useful to perform a structural analysis of the mutated proteins for establishing structural/functional relationships between the localization of a pathological mutation and the real need to administer a specific small molecule/cofactor/vitamin to a patient.

## 4. Small Molecules, Vitamins and Cofactors Administered as Single Molecules or as a Cocktail to Patients Affected by MD

Table 1 and Table 2 list the most common vitamins used in patients affected by MD as single molecules or as an antioxidant cocktail, with dietary reference intakes (DRIs, as reported in https://www.ncbi.nlm.nih.gov/books/NBK56068/table/summarytables.t7/?report=objectonly; accessed on 8 April 2022), whereas Table 3 and Table 4 list vitamin-related cofactors and the known associated protein targets.

### 4.1. Vitamin B1—Thiamine

**Figure 1:**
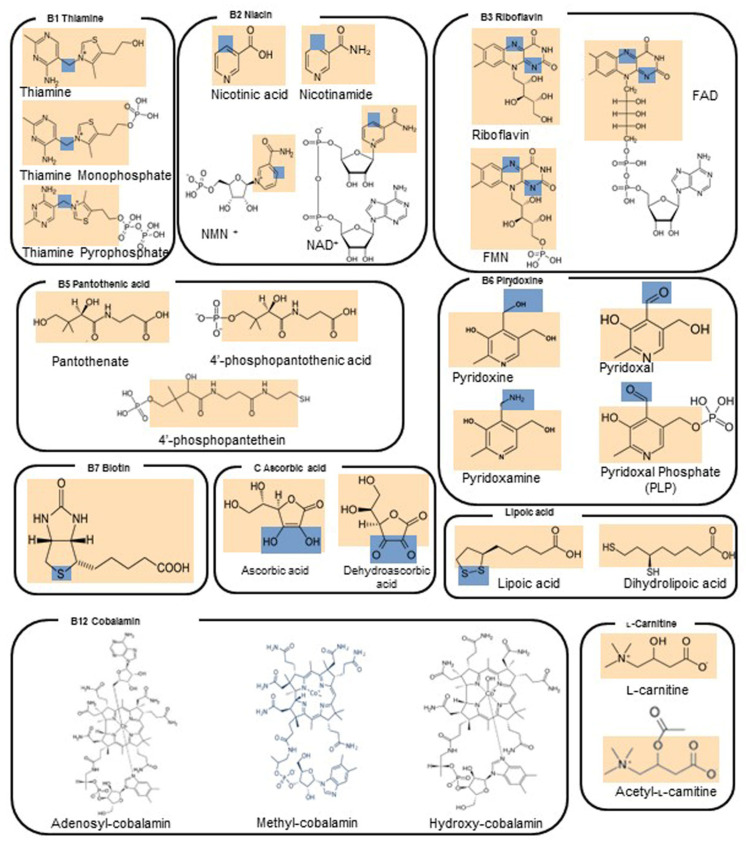

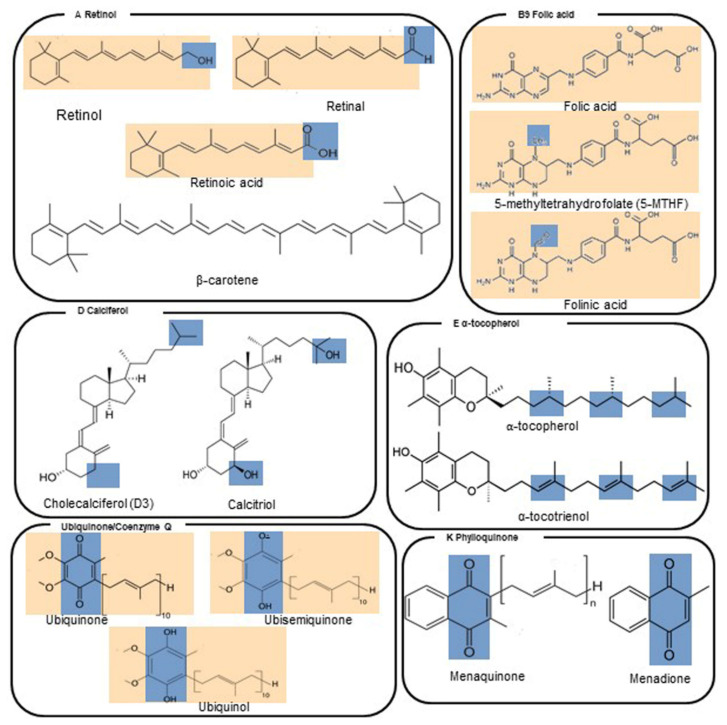
2D structures of vitamins and derived enzyme cofactors. Orange squares indicate the vitamin portion of each reported molecule. Blue squares indicate the reactive centers involved in electron transfer and/or redox changes.

**Figure 2:**
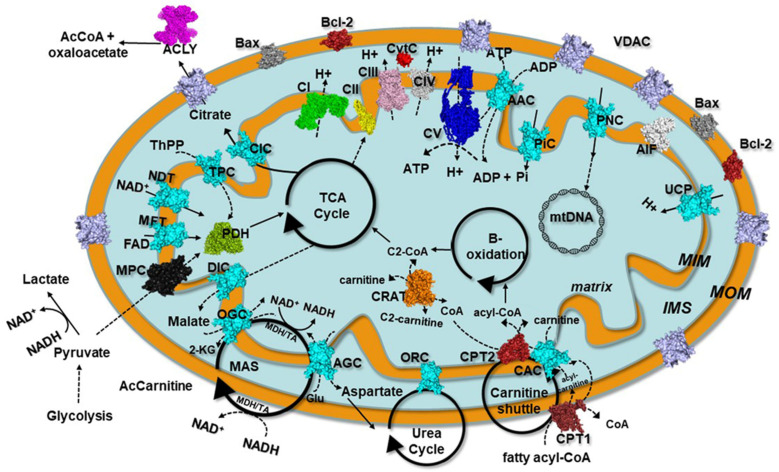
Scheme of a mitochondrion with a set of representative proteins, pathways and cycles. Respiratory chain complexes, mitochondrial transporters and other proteins are reported in surf representation and labeled. ATP synthase (CV) is reported in blue. Mitochondrial carriers are reported in cyan. VDAC is reported in pink. Bax and Bak/Bcl-2 are reported in dark grey and firebrick, respectively. MPC is reported in black. Complex I (CI), complex II (CII), complex III (CIII) and complex IV (CIV) are reported in green, yellow, magenta and grey, respectively. Black circular arrows indicate cyclic pathways. Red arrows indicate impaired pathways or reactions. Black solid/dashed lines indicate the possible direction of the reported reactions. Abbreviations: MIM: mitochondrial inner membrane; MOM, mitochondrial outer membrane; IMS, intermembrane space; AAC, ADP/ATP carrier, coded in H. sapiens by SLC25A4, SLC25A5, SLC25A6, SLC25A31; TPC, thiamine pyrophosphate carrier, coded by SLC25A19; CAC, carnitine/acyl-carnitine carrier, coded by SLC25A20; ORC, ornithine carrier, coded by SLC25A15 (or SLC25A2); AGC, aspartate/glutamate carrier, coded by SLC25A12 and SLC25A13; DIC, dicarboxylate carrier, coded by SLC25A10; NDT, assumed to be the NAD^+^ carrier, coded by SLC25A51; MFT, assumed to be the FAD (folate/riboflavin) carrier, coded by SLC25A32; OGC, malate/2-oxoglutarate carrier, coded by SLC25A11; CIC, citrate carrier, coded by SLC25A1; PiC, phosphate carrier, coded by SLC25A3; MAS, malate/aspartate shuttle; TCA, tricarboxylic acid cycle; Bax, Bcl-2 associated X protein; Bak, Bcl-2 antagonist/killer-1; Bcl-2, B-cell lymphoma-2; MPC, mitochondrial pyruvate carrier; UCP, uncoupling protein, coded by SLC25A7, SLC25A8, SLC25A9, SLC25A14, SLC25A27 and SLC25A30; CypD, cyclophilin D; CytC, cytochrome C; VDAC, voltage-dependent anion channel; AIF, apoptosis-inducing factor; PNC, pyrimidine nucleotide carrier, coded in H. sapiens by SLC25A33 and SLC25A36.

Thiamine is a water-soluble B vitamin (B1) (Figure 1) and is the precursor of thiamine monophosphate and thiamine pyrophosphate [40]. The ingested thiamin from food and dietary supplements is absorbed by the small intestine through active transport at nutritional doses and by passive diffusion at pharmacological doses. Most dietary thiamin is in phosphorylated forms, and intestinal phosphatases hydrolyze them to free thiamin before the vitamin is absorbed. The remaining dietary thiamin is in the free (absorbable) form [41]. Thiamine pyrophosphate enters mitochondria through a mitochondrial carrier coded by the nuclear SLC25A19 [42,43] (Figure 2). It is also used as a cofactor by several enzymes (Table 3) crucial for mitochondrial function and cell viability, such as pyruvate dehydrogenase and 2-oxoglutarate dehydrogenase, which are key enzymes of citrate metabolic pathways [44,45,46], where they are involved in the oxidation of pyruvate and 2-oxoglutarate [47].

Thiamine has been used in mitochondrial diseases as a single molecule or in combination with other antioxidants and drugs [39,40]. It is reported that thiamine administration to patients affected by MELAS syndrome and/or thiamine deficiency improves lactic acidosis and myopathy [48]. The administration of thiamine, in combination with coenzyme Q_10_ (CoQ_10_), carnitine and vitamins C and E, produced a clinical improvement in a Leigh patient with a severe encephalopathy [39,49] and in a recently described case of MEGDEL syndrome [50]. Mutations in the *SLC19A3* gene (encoding a ubiquitously expressed transmembrane thiamine transporter) are responsible for thiamine deficiency, which can manifest with encephalopathy, features of Leigh syndrome on neuroimaging and lactic acidosis. Similarly, mutations in the *SLC25A19* gene coding for a mitochondrial thiamine-pyrophosphate transporter of the inner mitochondrial membrane are responsible for a rare disease characterized by microcephaly and bilateral striatal necrosis [42,43,51]. The doses of thiamine for adults and children are reported in Table 1 [52]. Higher doses of thiamine (20 mg/kg/day) are administered to patients affected by SLC19A3 and SLC25A19 deficiencies to limit neurological and biochemical abnormalities [53].

### 4.2. Vitamin B2—Riboflavin

Riboflavin is a water-soluble vitamin also known as vitamin B_2_ and represents the precursor of flavin mononucleotide (FMN) and flavin adenine dinucleotide (FAD) (Figure 1). More than 90% of dietary riboflavin is in the form of FAD or FMN; the remaining 10% is made up of the free form and glycosides or esters. Most riboflavin is absorbed in the proximal small intestine. The body absorbs little riboflavin from single doses beyond 27 mg and stores only small amounts of riboflavin in the liver, heart, and kidneys. When excess amounts are consumed, they are either not absorbed or excreted in the urine.

Bacteria in the large intestine produce free riboflavin that can be absorbed by the large intestine in amounts depending on the diet. More riboflavin is produced after ingestion of vegetable-based than meat-based foods. It is known that riboflavin and/or FAD can enter mitochondria through a mitochondrial carrier coded by the nuclear gene SLC25A32 [42,43] (Figure 2). FMN is an important cofactor in complex I whereas FAD is a key cofactor of complex II, FAD synthase, dihydrolipoamide dehydrogenase, and pyruvate dehydrogenase, playing a crucial role in mitochondrial respiratory chain and Krebs cycle [44,54,55,56,57,58]. Riboflavin derivatives serve as a cofactor in several other key enzymatic reactions (Table 1 and Table 3) involving fatty acid oxidation [59] and pyrimidine biosynthesis [60]. FAD plays also a crucial role in the apoptosis-inducing factor, thioredoxin reductase and glutathione reductase [54].

Riboflavin has been used in mitochondrial diseases as a single molecule or in combination with other antioxidants and drugs [39,40,52,55,61,62,63]. It is reported that riboflavin supplementation to patients affected by MDs, with specific references to those caused by complex I and/or complex II mutations, improves lactic acidosis, myopathy and seizures [55,61,62]. The administration of riboflavin produced the amelioration of symptoms in patients affected by multiple acyl CoA dehydrogenase deficiency (MADD) caused by mutations at the electron-transport flavoprotein dehydrogenase (*ETFDH*) or in the electron transfer flavoprotein (*ETF*) consisting of α and β subunits encoded by *ETFA* and *ETFB*, respectively [64,65,66].

### 4.3. Vitamin B3—Niacin

Niacin or nicotinamide is a water-soluble vitamin also known as vitamin B_3_, and it represents the precursor of nicotinamide mononucleotide (NMN) and nicotinamide adenine dinucleotide (NAD^+^) (Figure 1). The ingested niacin is absorbed primarily in the small intestine, but some is absorbed in the stomach. All tissues in the body convert the absorbed niacin into its main metabolically active form, the coenzyme NAD^+^, which in turn is converted into another active form, the coenzyme nicotinamide adenine dinucleotide phosphate (NADP^+^), in all tissues except skeletal muscle. Most dietary niacin is in the form of nicotinic acid and nicotinamide, but some foods contain small amounts of NAD^+^ and NADP^+^. Cells are also able to convert tryptophan to NAD^+^, so tryptophan is considered a dietary source of niacin. When NAD^+^ and NADP^+^ are consumed in foods, they are converted to nicotinamide in the gut and then absorbed. 

It is known that reducing equivalents cross the inner mitochondrial membrane by using the malate/aspartate shuttle [67,68,69,70] to participate in the oxidation of the cytosolic NADH (Figure 2). In addition, it was recently proposed that SLC25A51 is responsible for the NAD^+^ uptake into mitochondria [71,72,73], although a biochemical assay with the purified SLC25A51 reconstituted into proteoliposomes, as done for other mitochondrial carriers [74], is still lacking and is required to determine kinetic parameters of mitochondrial NAD^+^ uptake. NMN is an important cofactor in mitochondrial 5′-(3′)-deoxyribonucleotidase, whereas NAD^+^ is a key cofactor of complex I, dihydrolipoamide dehydrogenase and pyruvate dehydrogenase, playing a crucial role in the mitochondrial respiratory chain and citrate metabolism [44,55,75]. NAD^+^ serves as a cofactor in several other key enzymatic reactions (Table 1 and Table 3) involving fatty acid oxidation [59] and pyrimidine biosynthesis [60]. NAD^+^ is also a crucial cofactor for the apoptosis-inducing factor (AIF), glutathione reductase and thioredoxin reductase protein [54], as well as for sirtuin deacetylases, which play an important role in neurodegeneration [76]. 

Niacin and niacin derivatives have been used in the treatment of mitochondrial diseases as single molecules or in combination with other antioxidants and drugs [39,49]. Niacin supplementation in patients affected by MDs, with specific references to those caused by complex I mutations, has been reported to improve lactic acidosis, myopathy and seizures [77], although previous papers have described niacin toxicity [78]. The administration of niacin is supposed to produce the amelioration of symptoms in patients affected by Leigh syndrome associated with mutations of nuclear [12,21,22] and mitochondrial genes [15,77]. Niacin is available in multivitamin–mineral products, in supplements containing other B-complex vitamins and in supplements containing niacin only. Nicotinic acid and nicotinamide are the two most common forms of niacin in supplements. Some niacin-only supplements contain 500 mg or more per serving, which is much higher than the recommended dietary allowance (RDA) for this nutrient. The doses of niacin for adults and children are reported in Table 1 [49,52].

### 4.4. Vitamin B5—Pantothenic Acid

Pantothenic acid, also known as vitamin B5 (Figure 1), is a water-soluble vitamin, widely distributed in foodstuffs and ubiquitous in nature [79]. About 85% of dietary pantothenic acid is in the form of CoA or phosphopantetheine. These forms are converted to pantothenic acid by digestive enzymes in the intestinal lumen and intestinal cells. Pantothenic acid is absorbed in the intestine and delivered directly into the bloodstream by active transport (and possibly simple diffusion at higher doses). Pantetheine, the dephosphorylated form of phosphopantetheine, however, is first taken up by intestinal cells and converted to pantothenic acid before being delivered into the bloodstream. The intestinal flora also produces pantothenic acid, but its contribution to the total amount of pantothenic acid that the body absorbs is not known. Red blood cells carry pantothenic acid throughout the body.

Within the cytosol, pantothenic acid is phosphorylated into 4′-phosphopantothenic acid by a pantothenate kinase (PANK). A derivative of the 4′-phosphopantothenic acid, 4′-phosphopantetheine, is transported into the mitochondria for the synthesis of CoA. Pantothenate is the key precursor for the biosynthesis of CoA.

Noteworthily, the acyl-carrier protein (ACP) requires 4-phosphopantothenic acid for its activity as a prosthetic group [79]. As a component of ACP and in the form of CoA, pantothenic acid is essential for several biochemical pathways involving the metabolism of lipids, proteins and carbohydrates and for mitochondrial energy production and respiration. 

Evidence from limited studies suggests that pantothenic acid might improve the heading process of skin wounds. Little or no toxicity has been associated with dietary or supplemental pantothenic acid intake. 

Pantothenic acid has been used in patients with Leber’s hereditary optic neuropathy (LHON), chronic progressive external ophthalmoplegia (CPEO), MELAS, neurogenic weakness, ataxia, retinitis pigmentosa or cytochrome c oxidase deficiency in combination with carnitine, CoQ_10_ and other vitamins. The cocktail resulted in increased ATP production and slower progression of clinical symptoms [39,80].

### 4.5. Vitamin B6—Pyridoxine

Pyridoxine (Vitamin B6) is a water-soluble vitamin (Figure 1) commonly found in fruits, vegetables and grains. Substantial proportions of pyridoxine from foods exist in glycosylated forms that exhibit reduced bioavailability. It must be obtained from the diet because the body cannot synthesize it [81].

The human body absorbs vitamin B6 in the jejunum. Phosphorylated forms of the vitamin are dephosphorylated, and the pool of free vitamin B6 is absorbed by passive diffusion.

The absorption of vitamin B6 from supplements is similar to that from food sources and does not differ substantially among the various forms of supplements. Although the body absorbs large pharmacological doses of vitamin B6 well, it quickly eliminates most of the vitamin in the urine. Pyridoxine can permeate the mitochondrial membranes by simple diffusion; however, the limited availability of pyridoxine and the lack of a pyridoxine-processing salvage enzyme in the mitochondrial matrix makes the pyridoxine mitochondrial uptake unlikely to be biologically important. Pyridoxine is metabolized to pyridoxal phosphate (PLP), essential for the synthesis of serotonin, norepinephrine, amino acids, aminolevulinic acid, lipids and carbohydrates. In contrast to pyridoxine, the PLP uptake is biologically important. PLP enters the mitochondrial matrix in a concentrative process that is insensitive to inhibitors and uncouplers of oxidative phosphorylation. PLP is a cofactor of several mitochondrial enzymes, such as synthases, ligases, aminotransferases, hydroxymethyltransferases and desulfurases [82]. Pyridoxine was used in combination with other vitamins of complex B, carnitine, CoQ_10_, vitamin C and vitamin K1 to treat patients with LHON, CPEO, MELAS, neuropathy, ataxia and retinitis pigmentosa (NARP) or cytochrome c oxidase deficiency [39,49,63].

### 4.6. Vitamin B7—Biotin

Biotin, also known as B7 vitamin (or vitamin H), is a water-soluble vitamin (Figure 1) naturally present in some foods and works as a covalently linked prosthetic group in metabolic enzymes [83]. Most biotin in foods is bound to protein, although some dietary biotin is in the free form. Gastrointestinal proteases and peptidases break down the protein-bound forms of ingested biotin into biocytin and biotin-oligopeptides, which undergo further processing by the enzyme biotinidase in the intestinal lumen able to release free biotin. The latter is then absorbed in the small intestine, and most biotin is stored in the liver.

The absorption rate of oral, free biotin is 100%, even when people consume pharmacological doses of biotin, up to 20 mg/day.

Biotin is a cofactor of five biotin-dependent carboxylases (BDCs), four of which are located primarily in the mitochondria, and plays a key role in the metabolism of fatty acids, glucose and amino acids [84]. BDCs supply intermediates for the tricarboxylic acid cycle, which are regularly removed for the synthesis of key metabolites such as heme or amino acids [85].

It is important to note that biotin-thiamine-responsive basal ganglia disease (BTBGD), an autosomal recessive disorder that results in severe neurological impairment, can mimic Leigh Syndrome and responds to biotin and thiamine supplementation [83,84,86,87]. Biotin has also been used in cocktails with other B vitamins [83,84,86].

### 4.7. Vitamin B9—Folic Acid

Folic acid (also known as vitamin B9) is a hydrophilic molecule (Figure 1) and is the generic term for a family of compounds that act as coenzymes in the folate cycle, which plays an important role in creatine synthesis, homocysteine re-methylation and DNA and RNA synthesis [83]. Food folates are hydrolyzed to the monoglutamate form in the gut before absorption by active transport across the intestinal mucosa. 

Folic acid is commonly present in foods and in dietary supplements. Passive diffusion also occurs when pharmacological doses of folic acid are consumed. Before folic acid enters the bloodstream, the enzyme dihydrofolate reductase reduces the monoglutamate form to tetrahydrofolate (THF) and converts it to either methyl or formyl forms. The main form of folate in plasma is 5-methyl-THF (5-MTHF).

Vitamin B9 can be found in different forms such as folinic acid and folic acid. Though folinic acid is chemically different from folic acid, they act in a similar way. Folic acid is reduced by a cascade of enzymatic reactions into its active form 5-MTHF. 5-MTHF is believed to be one of the central methyl donors required for mitochondrial protein and nucleotide and/or purine biosynthesis [88]. 

In contrast to folic acid, folinic acid is an immediate precursor of 5-MTHF. It is essential for the treatment of patients with primary or secondary cerebral folate deficiency (CFD), and its deficiency is associated with several primary mitochondrial disorders. Folinic acid determines the direct increase in brain 5-MTHF levels, believed to reduce white matter demyelination in patients with Kearns–Sayre syndrome (KSS). Limited evidence suggests that folinic acid might reverse the pathological condition associated with folic acid deficiency as in KSS. No adverse reactions were observed in patients with KSS after folinic acid supplementation, according to the dosage indicated in Table 1. However, hypersensitivity to folinic acid includes urticarial and anaphylactoid reactions [89].

### 4.8. Vitamin B12—Cobalamin

Cobalamin (Cbl), also known as vitamin B12, is a water-soluble vitamin (Figure 1) involved in DNA synthesis and fatty acid and amino acid metabolism. Vitamin B12 is bound to proteins in food and must be released before being absorbed. The process starts in the mouth when food is mixed with saliva. A high amount of vitamin B12 is released from its food matrix by the activity of hydrochloric acid and gastric proteases in the stomach, where it then binds to haptocorrin. In the duodenum, digestive enzymes free the vitamin B12 from haptocorrin, and this freed vitamin B12 combines with an intrinsic factor, a transport and delivery binding protein secreted by the stomach’s parietal cells. The resulting complex is absorbed in the distal ileum by receptor-mediated endocytosis. 

If vitamin B12 is added to fortified foods and dietary supplements, it is already in free form and therefore does not require the separation step. Cbl is involved in one-carbon transfer pathways. More in detail, Cbl participates in the methionine synthesis reaction (a cysteine source of glutathione biosynthesis) and in malonic acid accumulation [88]. There are three forms of Cbl in our diet. Adenosyl-cobalamin, methyl-cobalamin and hydroxy-cobalamin are found in fish, dairy products and organ meats. In the mitochondria, adenosyl-cobalamin is required for the synthesis of succinyl-CoA, an important intermediate of the tricarboxylic acid (TCA) cycle. Different types of Cbl-altered metabolic pathways are described, and according to the type of Cbl metabolic disruption, they are responsible for methylmalonic acidemia and/or homocystinuria [90]. Vitamin B12 deficiency treatment is based on vitamin hydroxocobalamin (OH-B12) supplementation. Recommended dietary intake of cobalamin was set at 0.002–0.003 mg/day, and vegetarians usually need to supplement Cbl in the diet [88]. 

### 4.9. Vitamin C—L-Ascorbic Acid

L-Ascorbic acid, also known as Vitamin C, is a water-soluble vitamin (Figure 1) working as a cofactor of several biochemical reactions. Indeed, ascorbic acid plays a crucial role in the biosynthesis of collagen, L-carnitine and neurotransmitters and/or in general in protein metabolism [49,63,91]. Vitamin C is naturally present in foods such as fruits and vegetables, and it is available as a dietary supplement. At high intake concentration, vitamin C causes diarrhea, nausea and other gastrointestinal disorders due to its osmotic effect [49,63,91]. The intestinal absorption of vitamin C is regulated by at least one specific dose-dependent, active transporter, and more in general vitamin C uptake is regulated by different transport pathways, under physiological conditions, which may be altered by aging and disease [92]. Approximately 70–90% of vitamin C is absorbed at moderate intakes of 30–180 mg/day. However, at doses above 1 g/day, absorption falls to less than 50%, and absorbed, unmetabolized ascorbic acid is excreted in the urine [93]. Results from pharmacokinetic studies indicate that oral doses of 1.25 g/day ascorbic acid produce mean peak plasma vitamin C concentrations of 135 micromol/L, which are about two times higher than those produced by consuming 200–300 mg/day of ascorbic acid from vitamin C-rich foods [94].

Dehydroascorbic acid, which is an oxidized form of vitamin C, enters mitochondria via facilitative glucose transporter 1 (GLUT1) and is reduced and accumulated as ascorbic acid [91]. Ascorbic acid has been used, in combination with vitamin K, to bypass complex III deficiency in the mitochondrial respiration process [49,63,91]. 

### 4.10. Lipoic Acid

Lipoic acid, also known as α-lipoic acid or thioctic acid (Figure 1), is an essential cofactor for the mitochondrial pyruvate dehydrogenase and 2-oxoglutarate dehydrogenase involved in the ATP production. It can also act as a scavenger of reactive oxygen species [63,95,96]. Lipoic acid is synthetized in the mitochondria from octanoic acid. In addition, lipoic acid is also retrieved from foods, such as meats, liver, fruits and vegetables, and it is accumulated in many tissues [63,95,96]. Lipoic acid was used in combination with coenzyme Q_10_ (CoQ_10_) and creatine to treat patients with mitochondrial myopathy, encephalopathy, mitochondrial neuro-gastrointestinal encephalopathy and other MDs [63,95,96]. The supplementary dietary intake seems to be well tolerated in clinical trials [63,95,96].

### 4.11. Vitamin A—Retinol

Vitamin A is a fat-soluble vitamin (Figure 1) essential for several physiological processes [97]. The human diet contains two sources of vitamin A: preformed vitamin A in foods from animal sources (retinol and retinyl esters) and provitamin A carotenoids in foods from vegetable sources (beta-carotene, alpha-carotene and beta-cryptoxanthin). The various forms of vitamin A are solubilized into micelles in the intestinal lumen and absorbed by duodenal mucosal cells. Retinyl esters and provitamin A carotenoids are converted to retinol after uptake into the lumen (for retinyl esters) or absorption (for provitamin A carotenoids). The absorption of preformed vitamin A esters from dietary supplements is 70–90%, and that of beta-carotene ranges from 8.7% to 65%. The deprivation of vitamin A determines an increase in reactive oxygen species (ROS) production, which causes mitochondrial membrane depolarization and rapid loss of plasma membrane integrity. The term vitamin A includes provitamin A carotenoids that are dietary precursors of retinol [98]. The RDA for men and women is 900 and 700 µg retinol activity equivalent per day, respectively. The tolerable upper intake level for adults is set at 3000 µg/day of preformed vitamin A [98]. The vitamin A deprivation causes the activation of poly-(ADP-ribose) polymerase 1 and catalyzes the NAD^+^ dependent synthesis and attachment of ADP-ribose units to gamma-carboxyl groups of glutamine residues of acceptor proteins. Chronic vitamin A intake causes a reduction in bone mineral density, teratogenicity and liver abnormalities [98]. More in detail, vitamin A intake induces cell fusion, hemolysis, swelling of mitochondria and lipid peroxidation in vitro [99]. It is known that retinol can induce cytochrome c release and an increase in the production of superoxide radical anion, although the right amount of retinol is essential for cell signaling [97,98,99].

### 4.12. Vitamin D—Calciferol

Vitamin D is a fat-soluble vitamin (Figure 1) that provides calcium and phosphorus levels necessary for the mineralization of bone tissue [100]. Vitamin D obtained from sun exposure, foods and supplements is biologically inert and must undergo two hydroxylation events in the body for activation [101]. In foods and dietary supplements, vitamin D has two main forms, D2 (ergocalciferol) and D3 (cholecalciferol), that differ chemically only in their side-chain structures. Both forms are well absorbed in the small intestine. Absorption occurs by simple passive diffusion and by a mechanism that involves intestinal membrane carrier proteins. The concurrent presence of fat in the gut enhances vitamin D absorption, but some vitamin D is absorbed even without dietary fat. Neither aging nor obesity alters vitamin D absorption from the gut. The 1,25-dihydroxyvitamin D, the active form of vitamin D3, is essential for several physiological functions, based on the correct mitochondrial respiratory function, including the control of the systemic inflammation and immune response [100,102,103]. Consequently, hypovitaminosis D impairs mitochondrial functions and enhances systemic inflammation and oxidative stress [100]. Vitamin D has antioxidant properties contributing to mitochondrial redox homeostasis. Thus, it is expected that vitamin D3 supplementation should allow keeping many degenerative disease processes under control [100,102,103]. 

### 4.13. Vitamin E—α-Tocopherol

Vitamin E, also known as α-tocopherol, is a major lipid-soluble chain-breaking antioxidant (Figure 1), scavenging lipid peroxyl radicals in a lipid milieu, and it is expected to exert an antioxidant effect when taken as a supplement [63,104]. One of the most important targets of vitamin E is the mitochondria [104,105]. The form of α-tocopherol present in food is *RRR*-α-tocopherol. The 2R-stereoisomeric forms of α-tocopherol (*RRR*-, *RRS*-, *RSR*- and *RSS*-α-tocopherol) are found in supplements. All forms are absorbed in the small intestine (passive diffusion). Vitamin E has been used as a supplement in the diet of patients affected by MDs, in combination with other antioxidants [49,63,104,105]. Although vitamin E is generally not considered toxic and its supplementation was even associated with a reduced risk of amyotrophic lateral sclerosis [106], the continued administration of this vitamin was associated with an increased risk of hemorrhage, particularly in anticoagulant-treated patients [107].

### 4.14. Vitamin K—Phylloquinone

Phylloquinone, also known as Vitamin K, is a fat-liposoluble vitamin (Figure 1) with antioxidant properties, used as a cofactor for enzymes involved in blood clotting and bone metabolism [108]. Phylloquinone is present primarily in green leafy vegetables and is the main dietary form of vitamin K. Menaquinones are also produced by bacteria in the human gut [109]. Vitamin K is incorporated into mixed micelles via the action of bile and pancreatic enzymes, and it is absorbed by enterocytes of the small intestine. The absorption rate of phylloquinone in its free form is approximately 80%, but its absorption rate from foods is significantly lower. Several forms of vitamin K are used in dietary supplements, including vitamin K1 as phylloquinone or phytonadione (a synthetic form of vitamin K1) and vitamin K2 as MK-4 or MK-7. Few data are available on the relative bioavailability of the various forms of vitamin K supplements. One study found that both phytonadione and MK-7 supplements are well absorbed, but MK-7 has a longer half-life.

Menadione, which is sometimes called vitamin K3, is another synthetic form of vitamin K. It was shown to damage hepatic cells, so it is no longer used in dietary supplements or fortified foods. Vitamin K shows very low toxicity levels also in the case of long-term supplementations [110]. Phylloquinone was used in combination with other vitamins or drugs for the treatment of primary mitochondrial disorders [63,111,112,113], although it cannot replace coenzyme Q10 in the mitochondrial respiratory chain of mammalian cells [114], despite what was observed in drosophila models [115].

### 4.15. Coenzyme Q_10_ (Reduced, as Ubiquinol; Oxidized, as Ubiquinone)

**Figure 3:**
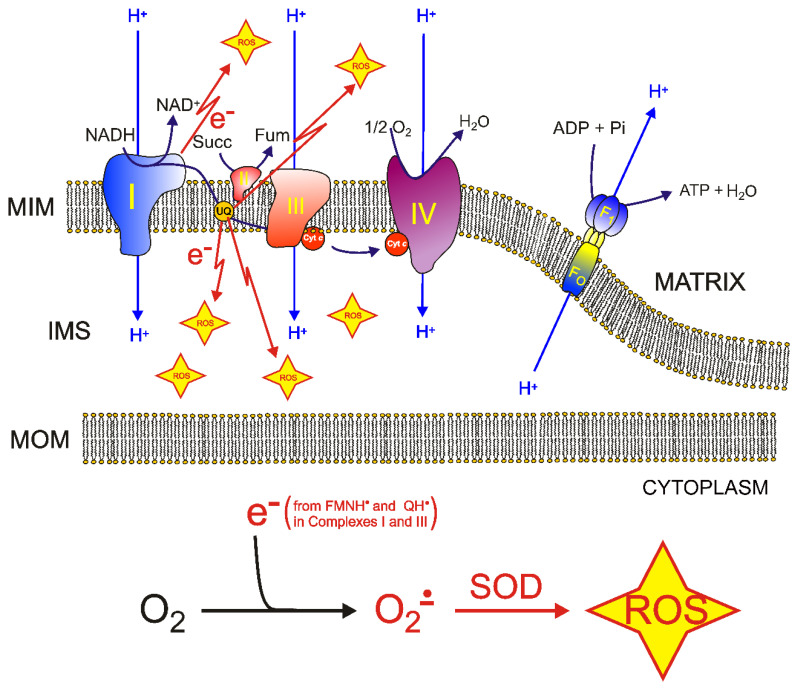
Mitochondrial respiratory chain and sites of superoxide production within mitochondria. Incomplete reduction of O_2_ at complex I and complex III during mitochondrial respiration leads to the formation of the superoxide anion. The superoxide anion produced at the level of complex I is released into the mitochondrial matrix. The superoxide anion produced at the level of complex III can be released either to the matrix space or to the intermembrane space. MIM and MOM stand for mitochondrial inner and outer membrane, respectively. IMS stands for intermembrane space.

CoQ_10_ is a cofactor (Figure 1) endogenously synthesized within mammalian mitochondria and is an electron transporter crucial for the function of the mitochondrial respiratory chain (Figure 3), shuttling electrons from complex I, complex II and glycerol-3P-dehydrogenase and from several other enzymes, such as those involved in beta-oxidation or citrate metabolic pathways [39,52,63,75,116,117].

CoQ_10_ also appears to play an important role in the function of other dehydrogenases (i.e., AIF, glutathione reductase and thioredoxin reductase [54,118,119]). CoQ_10_ plays an important intracellular signaling role and serves as both an antioxidant and pro-oxidant molecule, being able to serve as an oxygen radical scavenger [39,52,116,117]. CoQ_10_ modulates the mitochondrial permeability transition pore in the regulation of mitochondrial apoptosis [39,52,116,117]. 

CoQ_10_ supplementation is used in the treatment of patients affected by mitochondrial diseases [39,52,116,117] depending on defects of the mitochondrial respiratory chain independently from the phenotype and is specifically employed in primary coenzyme Q_10_ deficiency, manifesting with different phenotypes, such as encephalomyopathy (Leigh or Leigh-like syndrome), cerebellar ataxia, severe infantile multisystemic disease, nephropathy and myopathy [12,21,39,63,120,121,122]. 

Patients with primary CoQ_10_ deficiency and the myopathic variant have clinical manifestations affecting both the peripheral and central nervous systems: severe exercise intolerance, peripheral neuropathy, seizures, ataxia, pyramidal signs and mental retardation. Muscle biopsies show abundant RRF and increased lipids. Muscle CoQ_10_ level is reduced. Primary CoQ deficiencies may respond to exogenous CoQ_10_ administration with a general amelioration of the patient clinical conditions [39,63,86,116,123,124,125,126,127]. CoQ_10_ supplementation is particularly effective in improving symptoms in the myopathies associated with pathogenic variants in ETFDH [64,65,66].

CoQ_10_ is insoluble in water, and its powder formulations are very poorly absorbed from the intestine [128]. The conjugation of CoQ_10_ with nanoparticles in suspension has improved CoQ_10_ bioavailability [129]. The reduced CoQ_10_ as ubiquinol is 3 to 5 times better absorbed than its oxidized counterpart in the form of ubiquinone [39,49,63,130]. The doses of ubiquinone or ubiquinol for adults and children are reported in Table 1. High-dose oral CoQ_10_ supplementation (300–1500 mg daily) appears to be beneficial in all the investigated patients.

### 4.16. L-Carnitine/Acetyl-L-Carnitine

L-Carnitine is a small molecule structurally related to glutamate (Figure 1), playing a critical role in the process of mitochondrial β-oxidation of fatty acids and in the esterification of free fatty acids that may otherwise be sequestered by coenzyme A (CoA). Carnitine transfers long-chain fatty acids across the mitochondrial inner membrane as acylcarnitine esters through the carnitine/acyl-carnitine antiporter coded by SLC25A20 (Figure 2) [42,43,131]. These esters are oxidized to acetyl CoA, which enters the Krebs cycle and results in the subsequent generation of ATP via oxidative phosphorylation. Certain tissues, such as skeletal muscle, heart and liver, largely depend on β-oxidation for ATP production. Carnitine may prevent CoA depletion and remove excess, potentially toxic, acyl compounds. Diet is the source of 75% of carnitine, and 25% is synthesized in the body, predominantly within muscle, liver and kidneys. Skeletal muscles contain 90% of total body carnitine [131,132]. The plasma concentration of carnitine is regulated by its active reabsorption in the proximal renal tubules. Most (54–86%) dietary carnitine is absorbed in the small intestine and enters the bloodstream. The kidneys efficiently conserve carnitine. Rather than being metabolized, excess carnitine is excreted in the urine to maintain stable blood concentrations.

Primary carnitine deficiency due to defective carnitine synthesis or transport is not a typical feature of MDs. However, patients with respiratory chain defects tend to have lower than average free carnitine levels and increased esterified carnitine levels in plasma. This shift may reflect a partial β-oxidation impairment [117]. L-Carnitine supplementation for MDs is a common practice aimed at restoring free carnitine levels and removing accumulating toxic acyl compounds. It has been found that L-carnitine or acetyl-L-carnitine supplementation may stimulate mitochondrial biogenesis [133,134,135,136]. In MDs, carnitine is generally administered in combination with other vitamins [133,137], although a real benefit of the administration of carnitine in patients affected by MDs remains to be demonstrated with dedicated studies.

## 5. Molecular Mechanisms of ROS Production and Mitochondrial Damage Observed in Cells from Mitochondrial Patient Tissues

To support respiration, mitochondria consume 80–90% of the cell’s oxygen. It has been reported that approximately 0.2–2% of the oxygen taken up by the cell is transformed into ROS by mitochondrial respiration [138]. The main source of mitochondrial ROS production is the electron transport chain (ETC) at the level of complex I and complex III (Figure 3) [139,140]. The mitochondrial complex I can produce a superoxide anion, mainly when the matrix NADH/NAD^+^ ratio is high and when mitochondria are not producing ATP in the presence of a high proton-motive force and a reduced coenzyme Q pool [139]. Superoxide production at mitochondrial complex III is probably due to the unstable ubisemiquinone molecules [139,141] or cytochrome b [142]. 

The primary ROS produced by mitochondrial ETC is superoxide anion (O_2_^•−^) [139], which can be subsequently converted to hydrogen peroxide (H_2_O_2_) by spontaneous dismutation or by the action of the superoxide dismutase enzyme. Subsequently, hydrogen peroxide, in turn, can be converted into water by the glutathione peroxidase or catalase; otherwise, in the presence of divalent cations such as iron, H_2_O_2_ can undergo the Fenton reaction to produce hydroxyl radical (•OH), which can be more harmful to cellular components. 

ROS production is counteracted by an intricate antioxidant defense system. The balance between ROS production and antioxidant defenses determines the level of oxidative stress [143,144,145,146]. The consequences of this stress may include alterations of proteins, DNA and membrane phospholipids, mainly in the immediate cellular surroundings where these reactive species are produced.

Among mitochondrial membrane phospholipids, cardiolipin is particularly susceptible to ROS-induced oxidation, either due to its high content of unsaturated fatty acids or due to its location near the site of ROS production. It is well accepted that cardiolipin plays a pivotal role in several mitochondrial bioenergetic processes. In fact, cardiolipin has been reported to interact with several proteins and enzymes of the inner mitochondrial membrane involved in OXPHOS [12,42,43,46,68,69,147,148,149,150]. It has been proposed that in some physio-pathological conditions, ROS-induced oxidative damage to mitochondrial cardiolipin can increase electron leakage from respiratory chain complexes, producing more superoxide anions and triggering a cycle of ROS-induced mitochondrial membrane damage, which ultimately leads to alterations in mitochondrial bioenergetics [151,152].

## 6. The Need for a Structural Analysis for Evaluating the Real Need for Vitamin Supplementation

Vitamins are administered to patients affected by MDs with the expectation that these molecules will act as ROS scavengers and/or counteract ROS formation, also after targeting an impaired metabolic pathway or a specific protein complex or enzyme (Figure 2). If the damaged protein complex or the mutated enzymes are involved in oxidative reactions, the provided supplementation should rescue (at least partially) the affected protein function [39,49,63]. That is the case of respiratory chain dysfunction causing Leigh syndrome and Leigh-like syndrome, MERRF or MELAS, whose patients are often treated with complex I cofactor precursors (riboflavin and rarely niacin) and more in general with antioxidant cocktails (including CoQ_10_) [8,12,15,21,22,24,25,121,153]. 

However, respiratory chain complex impairment may be related to numerous/different genetic contexts. For example, respiratory chain complex I and IV deficiencies may be related to missense mutations of one of the subunits of the cited complexes, or to missense mutations of chaperone proteins or assembly factors involved in complex I and complex IV assembly and folding. In addition, respiratory chain complex defects causing a Leigh-like syndrome [121] may be related to mutations affecting enzymes not directly linked to complex I activity, as in the case of the complex I NDUFAF6 assembly factor [22], the mitochondrial matrix carnitine O-acetyltransferase (CRAT) [21] or the inner membrane mitochondrial transporter dicarboxylate carrier (DIC) [12], or even related to extra mitochondrial proteins, as for the serine active site-containing protein 1 (SERAC1), a protein essential for phosphatidylglycerol remodeling and mitochondrial function, whose mutations are associated with the MEGDEL syndrome [26] (Table 5). 

In the case of MELAS and MERRF syndromes, caused by mutations affecting two different mitochondrial tRNAs, the impairment of the OXPHOS system most likely depends on an incorrect folding and assembly of the two protein complexes due to the reduced availability of amino acids carried by the two tRNAs [25].

Currently, our technology does not allow the specific targeting of an impaired protein. We have also issues with the delivery of drugs to a specific tissue [154]. However, the indiscriminate administration of antioxidant cocktails could have potentially negative effects due to cell-signaling damage induced by off-target effects [155,156]. 

Thus, it would be reasonable to establish case by case if vitamin supplementation can be useful for rescuing an altered metabolic pathway or an impaired protein function. This approach would allow a more focused treatment, according to the affected protein and to the localization of the investigated specific mutation in the structure of the affected protein, eventually supported by biochemical investigations that prove a deficiency of the vitamins and cofactors to be administered.

## 7. Missense Mutations Responsible for Severe Encephalomyopathies: The Case of *MT-ND5*, *NDUFAF6* and *SERAC1*

**Figure 4:**
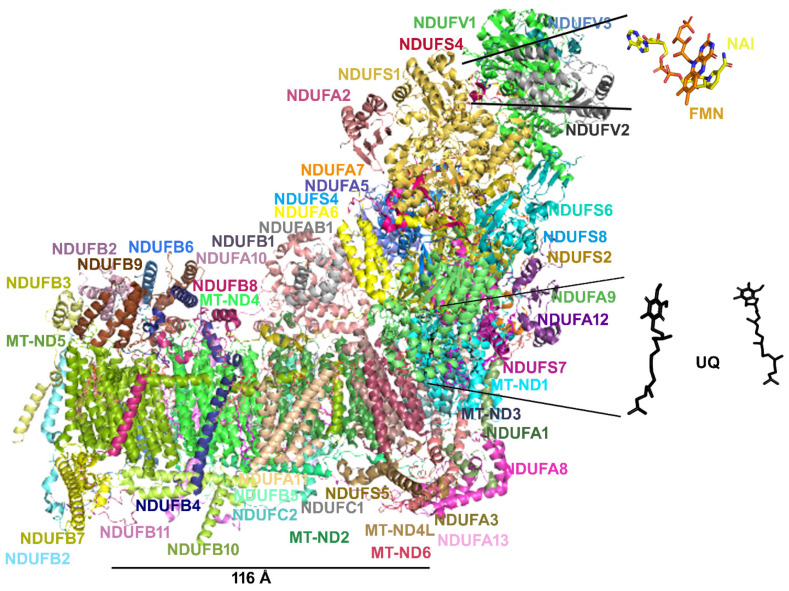
The sus complex I structure (7v2r.pdb, [157]) is reported in colored cartoon representation as a representative complex I structure from mammalian species. The same color scheme is used for the labels indicating each reported subunit. The subunits NUDFA7, NDUFC1 and NDUFB1 are not viewable in this side view. FMN and NAD^+^ are reported in orange and yellow sticks, as shown in the zoomed view, at the level of subunit NDUFV1. UQ is reported in black sticks as shown in the zoomed view, at the level of MT-ND1, NDUFS2, NDUFS7 and NDUFA9. The distance between the center of mass of ND5 and the center of mass of the closest ubiquinone molecule is represented by the black solid line, here used as a reference distance.

The mitochondrial DNA mutations m.13513G>A and m.13042G>A are among the most studied mitochondrial DNA mutations affecting complex I [157,158,159]. These mutations cause the amino acid replacement D393N and A236T, respectively, at the MT-ND5 subunit, determining a similar neurodegenerative phenotype [160]. For slowing down the neurodegeneration often observed in patients showing mutations at ND5, patients are treated with an antioxidant cocktail including thiamine derivatives, carnitine, ascorbate, pyridoxal phosphate, cobalamin, biotin, beyond niacin, riboflavin and CoQ_10_, known the latter three for being involved in direct interactions with complex I [157] (Figure 4). It appears that complex I does not bind thiamine derivatives, carnitine, ascorbate, pyridoxal phosphate, cobalamin and biotin analogs. Thus, it is questionable if the administration of those ligands can directly improve mitochondrial function or counteract ROS production through interactions with complex I. Notably, occasional ameliorative effects might be ascribed to a generic scavenger effect played by those molecules. Conversely, it should be stressed that the cited cofactors can target other proteins that support and/or depend on mitochondrial function; thus, it cannot be excluded that the administration of those molecules can even produce an overload of work for complex I, whose function is already partially damaged due to ND5 mutations.

According to the solved structure of complex I, the ND5 subunit is located at the end of the complex I membrane arm (Figure 4). It is known that the two investigated mutations can cause an alteration in the local secondary structure packing and an important change in the number and type of both short/medium interactions and long-range interactions that may affect the flexibility and packing of the local secondary structure elements [161,162]. In fact, the replacement of aspartic acid with an asparagine causes the loss of local ionic interactions and impairs the local net charge, whereas the replacement of the hydrophobic alanine with the polar threonine causes an increase in the number of local hydrophilic interactions and impairment in the local hydrophobic interaction network [161,162], which can cause problems in ND5 protein folding.

Notably, the investigated mutations at ND5 are very far from NADH or FMN binding regions (Figure 3). Thus, the administration of FMN (i.e., riboflavin) and NADH (i.e., niacin) precursors to patients carrying the m.13513G>A (D393N) or m.13042G>A (A236T) mutations also remains questionable, because these molecules could not help to make complex I more efficient, due to the fact that they interact with subunits far from ND5. 

Conversely, the administration of ubiquinol (CoQH2)/ubiquinone (CoQ) or α-tocopherol to a patient with the above-cited mutations could be more useful due to the similarity of the three molecules with the native coenzyme Q, which moves across the membrane arm of complex I and can somewhat participate in the proton translocation through ND5 subunit [49,63,117]. 

Notably, the administration of CoQH2 (i.e., the reduced form of coenzyme Q) could help in “overcoming” complex I inactivation by bypassing it [49,63,117].

It is instead expected that the administration of niacin (and NADH structurally related ligands) and riboflavin (and FMN or FAD structurally related ligands) can produce important results in patients carrying mutations in those complex subunits directly involved in interactions with NADH and FMN cofactors [157].

At variance with missense mutations directly affecting the activity of crucial complex I subunits, other proteins involved in the folding of respiratory chain complexes or participating in the same metabolic pathways can affect the activity of respiratory chain complexes, in case of mutations. As an example of proteins responsible for the impairment of mitochondrial respiration, we had the possibility to analyze in our laboratories the case of NDUFAF6, which plays a crucial role in the assembly of complex I [23] and SERAC1 responsible for the MEGDEL syndrome (3-methylglutaconic acidemia, deafness, encephalopathy and Leigh-like syndrome; https://www.omim.org/entry/614739, accessed on 8 April 2022), which shares with Leigh syndromes several phenotypic traits [163,164]. Notably, SERAC1 plays a crucial role in phospholipid remodeling at the interface between the endoplasmic reticulum and mitochondria. SERAC1 mutations impair mitochondrial respiration and intracellular cholesterol trafficking causing dystonia and deafness [164]. Conversely, mutations occurring at the NDUFAF6 assembly factor (e.g., I124T, A178P [22,165]) cause a neurodegenerative phenotype [23,166] similar to the one observed in NDUFAF1 or NDUFAF5 mutations characterized by severe degenerative encephalomyopathies [167,168]. The administration of antioxidant cocktails to patients affected by mutations at complex I assembly factors or by mutations affecting SERAC1 should be decided according to the demonstrated biochemical need of the cofactors to be administered. In the case of SERAC1 mutations, instead of antioxidant cocktails, one might consider the possibility of administering a supplement containing phosphatidylglycerol-34:1 and/or phosphatidylglycerol-36:1, both precursors of cardiolipin, important for the correct folding of mitochondrial membrane proteins. Indeed, the phosphatidylglycerol-34:1/phosphatidylglycerol-36:1 ratio appears to be unbalanced as a consequence of SERAC1 mutations [164]. Thus, it is expected that providing a diet supplement based on cardiolipin and/or phosphatidylglycerol lipids, or based on molecules able to stimulate cardiolipin and other phospholipid production, might help in slowing down mitochondria-mediated cell death and tissue damage in patients affected by the above-cited mutations, as observed in in vitro assays performed on model systems [123,169,170,171].

## 8. The Case of Carnitine O-Acetyltransferase (CRAT) Deficiency

**Figure 5:**
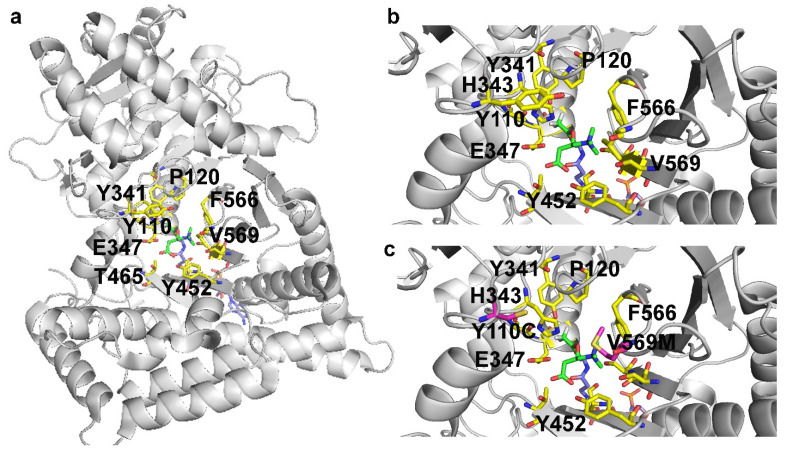
The side view of the CRAT enzyme is reported in grey cartoon representation. (**a**) Residues within 4 Å from carnitine (green sticks) are reported in yellow sticks. Acetyl-CoA is reported in blue sticks. The exploded views of the CRAT wild type and of the double mutant (Y110C/V69M) are reported in panel (**b**) and panel (**c**), respectively. The missense mutations Y110C and V569M are reported in magenta sticks in panel (**c**).

The CRAT enzyme catalyzes the reversible exchange of acetyl groups between CoA and carnitine, thus being involved in many metabolic pathways, including the ones essential for mitochondrial energy production [21]. Although CRAT deficiency has been observed in sporadic cases of mitochondrial encephalopathy for a long time [172,173], the genetic bases of this defect have been only recently proven [21]. This is the case of a Leigh syndrome patient, with respiratory chain deficiency in muscle but not in fibroblasts, who also showed two CRAT mutations (Y110C and V569M, Figure 5) that were demonstrated to cause the impairment of the enzymatic CRAT activity [21].

It was proposed that the respiratory chain defect observed in this patient might have been secondary to the excess of acetyl-CoA in mitochondria that accumulates when substrate oxidation exceeds the energy demand and that cannot be buffered by CRAT via acetylcarnitine, as physiologically occurs [174].

This patient was treated with a mix of cofactors aiming at limiting the excess ROS production. Although the initial lactic acidosis was reduced, it is unlikely that the mix of the above-cited cofactors rescued the activity of CRAT and/or of the respiratory chain complexes.

Most likely, coenzyme Q alone, in its reduced form could be somewhat more able to stimulate the complex III activity pushing the electron transfer regulated by the functional complex I.

Conversely, administrating carnitine to these patients might help in scavenging the excess acetyl-CoA by stimulating CRAT activity and by increasing the rate of spontaneous trans-acetylation reactions from CoA to carnitine, as well as by stimulating mitochondrial biogenesis if supplied together with lipoic acid [133,134,135]. 

## 9. SLC25A10 Dicarboxylate Carrier (DIC) Deficiency

Similarly to the patient affected by CRAT deficiency, another patient, affected by SLC25A10 deficiency, showed impaired activity of the respiratory complex I in muscle biopsy but not in fibroblasts, in addition to a multidrug-resistant degenerative myopathy and to a severe epileptic and progressive encephalopathy [12]. 

**Figure 6:**
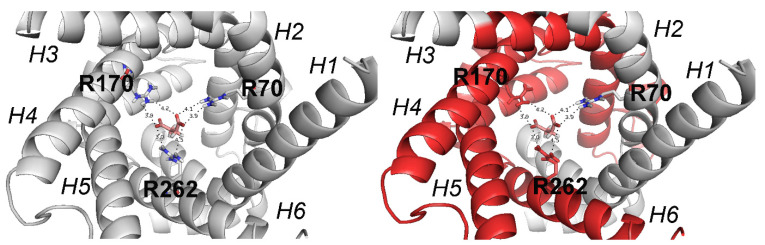
The structure of the SLC25A10 protein is reported in cartoon representation. The three arginine residues forming the three contact points of DIC, in the similarly located MC binding region shared by all eukaryotic organisms hosting mitochondria [43,176,177,178,179,180], are reported in stick representation and labeled. The red color in the cartoon representation in the right panel indicates the protein region (including transmembrane helices H3-H6) that is not translated due to the premature stop codon mutation.

The quantity and the activity of SLC25A10, which transports dicarboxylates (malate and succinate) and phosphate across the inner mitochondrial membrane, were almost completely absent in this patient due to mutations that caused both aberrant RNA splicing and the insertion of a premature stop codon abolishing the translation of 70% of the original protein sequence (Figure 6) [12].

Further, the patient’s cells were also depleted in the main antioxidant molecules NADPH and GSH [12], suggesting that SLC25A10 lack-of-function might have reduced the entrance of reducing equivalents into mitochondria via its substrates malate and succinate, thus conferring oxidative stress vulnerability and the impairment of mitochondrial respiration.

Thus, in this case, the possible beneficial effects provided by generic antioxidant cocktails, also including oxidized coenzyme Q, remain marginal [12]. Conversely, one might consider limiting the cellular damage by the administration of succinate-ester derivatives, or more generally succinate prodrugs [175], able to enter mitochondria independently from SLC25A10 (i.e., as in the case of the DIC patient hosting a truncated DIC protein at residue 102 out of 287 (Figure 6)), or the administration of ubiquinol, i.e., the reduced form of coenzyme Q (Figure 1), which directly donates electrons to the respiratory chain complex III, bypassing succinate/fumarate dehydrogenase.

## 10. MDs Depending on AA-tRNA Mutations or AA-tRNA Synthetase Mutations

**Figure 7:**
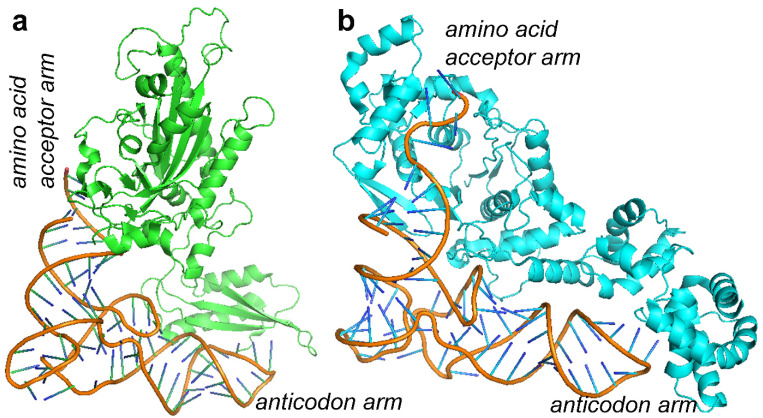
The mitochondrial Phe-tRNA synthetase (green cartoon) is reported in complex with Phe-tRNA (orange cartoon, green/blue sticks) in panel (**a**), whereas the cytosolic Glu-tRNA synthetase (cyan cartoon) is reported in complex with Glu-tRNA (orange cartoon, cyan/blue sticks) in panel (**b**). The positions of the amino acid acceptor arm and of the anticodon arm are indicated by labels.

Different forms of severe encephalopathies depend on mutated mitochondrial tRNA, as in the case of the mitochondrial tRNA-Lys (MERRF), tRNA-Leu (MELAS) or tRNA-Phe, and on an impaired tRNA synthetase (as for EARS2 deficiency, which depends on the mutated glutamyl-tRNA synthetase) [19,24,25,181,182]. The tRNAPhe from the crystallized human mitochondrial PheRS complexed with tRNAPhe in the active open state (3TUP.pdb, [183]) was already used to model a mutation of the mitochondrial tRNA-Phe (Figure 7) observed in a patient with a severe encephalopathy [24]. Notably, in the last 25 years, several AA-tRNA synthetases were crystallized in complex with AA-tRNAs (Table 6) [183,184,185,186,187,188,189,190,191,192,193,194,195,196,197,198,199], and they can be used to model the mutated mitochondrial tRNA-Lys and tRNA-Leu in complex with the related tRNA-AA synthetase to investigate the corresponding mitochondrial AA-tRNA/tRNA synthetase complexes impaired in MERRF, MELAS or EARS2 patients, for clarifying the mechanism of impaired amino acid supply to mitochondria for mitochondrial protein synthesis. The investigated pathogenic mutations appear to seriously affect complex IV activity, most likely due to incorrect folding of the same complex IV, due to the few available aa-loaded tRNAs within mitochondria [19,24,25,181,182]. 

Given that complex IV inactivation most likely depends on an unfolding problem, due to the missing AA-loaded-tRNA, patients affected by mitochondrial diseases caused by tRNA mutations or by mutations impairing the tRNA-AA synthetase might be treated with amino acids, or amino acids precursors, whose availability may be altered in specific tissues hosting the mutated tRNA or the corresponding synthetase, after having ascertained a clear deficiency of the small molecules to be administered. The aim of amino acid precursor administration is to partially compensate for the damaged ability in forming tRNA-AA with a greater availability of the amino acid analogs to stimulate the activity of the tRNA-AA synthetase. In such conditions, in case of problems with protein folding or assembly, it is expected that amino acid supplementation might be more successful than antioxidant administration. 

In this regard, a recent experimental treatment based on the administration of a polypeptide known as elamipretide gave encouraging, although very preliminary, results in 30 patients affected by primary mitochondrial myopathies [200]. However, the mechanism of action of elamipretide at the molecular level has not been completely clarified yet [126]. Indeed, it is known that elamipretide binds lipid bilayers and modulates surface electrostatics, but it remains to be understood if elamipretide supports the folding of membrane proteins also by interacting with chaperon proteins and protein complex assembly factors, beyond the described interactions with cardiolipin [201,202,203,204,205]. It was recently proposed that polypeptide-based therapies can also play a protective role by reducing the excess apoptosis observed in cell models and/or tissues from patients affected by mitochondrial diseases [146,203,206,207,208]. 

Conversely, a tissue-specific delivery of a synthetic AA-loaded-tRNA (also combined with specific cofactors/antioxidants) aiming to replace the impaired AA-tRNA/tRNA synthetase [209] might be attempted in the future. In general, the intranasal delivery of proteins and drugs should be considered as an option for slowing down neurodegeneration in MD patients more efficiently. Something similar was already successfully tested by intranasal administration of growth factors for treating other brain injuries [210,211,212]. In addition, new pieces of evidence report important advancements in the delivery of oligonucleotides and nucleic acids in general in several biological models [213,214], by using liposomes and/or specific peptides for mitochondrial delivery [215,216,217,218,219], coherently with the recent mRNA-based vaccine success [220,221] and advances in the analysis of mitochondrial tRNA import [222,223,224,225].

## 11. CoQ Analogs and Mitochondrial Delivery Systems: Organic/Inorganic Chemicals for Stimulating Mitochondrial Activity

**Figure 8:**
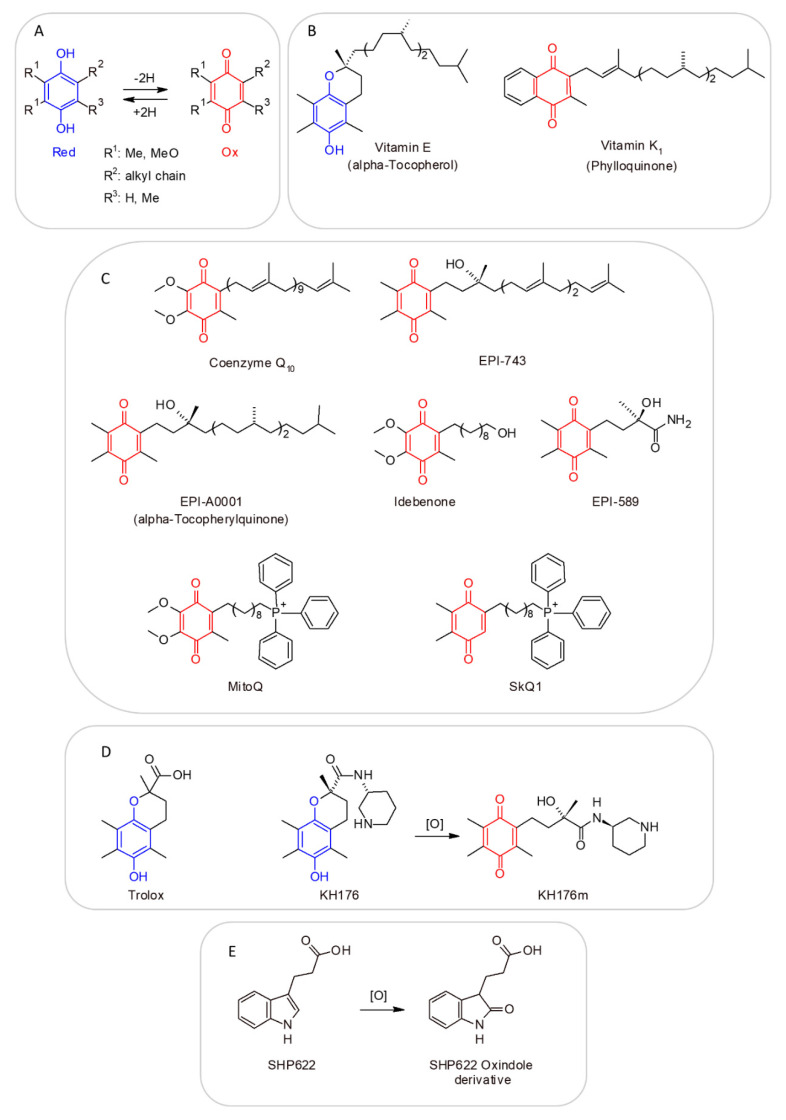
Chemical structures of compounds that are potentially useful as a pharmacological intervention for MDs. (**A**) General formula; (**B**) structures of vitamins E and K; (**C**) exemplary redox-active compounds; (**D**) KH176 as a Trolox analog and bioprecursor of KH176m; (**E**) SHP622, a peculiar antioxidant compound, and its oxidized product oxindole.

Numerous small molecules have been proposed as redox-active drugs, possibly useful as antioxidants, electron conductors, and/or pro-oxidants [39]. Most of those experimental drugs present a (hydro)quinone scaffold (general structure A, Figure 8), thus being closely related to CoQ_10_. On the other hand, the redox-active compounds reported so far display evident similarities to the antioxidant vitamins E (**α**-tocopherol) and K (phylloquinone—K_1_; Figure 8B). Generally, the ring scaffold is decorated with electron-donating substituents (R^1^ and R^3^; Figure 8C) while an alkyl chain (R^2^) is mainly responsible for ADME and targeting issues [226]. KH176 was developed as a Trolox analog and may be considered a bioprecursor of the active metabolite KH176m (Figure 8D) [227]. As a notable exception, SHP622 is not related to CoQ_10_ (Figure 8E). Interestingly, it may act as an electron donor generating the corresponding oxindole derivative. The latter cannot be directly reduced back to the parent compound. This is why SHP622 lacks pro-oxidant activity and, in principle, should not be considered a redox-active compound. 

Idebenone, EPI-743, KH176 and more recently all the other cited small molecules have been used in preclinical studies and then employed in clinics for counteracting the mitochondrial respiratory chain defects [86,123,124,125,126,127]. EPI-743, already used in clinical trials for the treatment of Leigh syndrome [228], is now being investigated in clinical trials for its potential usage in the treatment of visual and neurological complications due to a secondary altered redox state in Cbl C deficiency [229] and in Friedreich ataxia [230]. 

However, it is still not clear whether these molecules, beyond their ROS scavenger properties, exert their antioxidant activity by targeting a specific protein or a specific group of proteins. Both surface plasmon resonance interaction studies and in silico modeling indicated peroxiredoxin as a possible target of KH176m [227]. On the other hand, while CoQ analogs can stimulate mitochondrial respiratory chain activity, it is also possible that those small molecules can target other mitochondrial/cytosolic enzymes (using CoQ analogs as cofactors) and be involved in oxidative reactions [54,227,231,232]. 

The undesired targeting of other proteins can cause damage to the endogenous ROS signaling (off-target effects) that may result, in case of prolonged administration, in tissue damage [233,234,235]. 

According to these findings and observations, the administration of the above-reported molecules to MD-affected patients should be carefully evaluated case by case. The main scope of the administration of those molecules is indeed to counteract ROS increase (acting as ROS scavengers) and improve the activity of the impaired respiratory chain complexes to provide a valid therapeutic strategy for dealing with severe MDs. Conversely, the greater success of those molecules should be related to their possible direct involvement in the function of the affected protein. Thus, a therapeutical approach based on idebenone, EPI-743 and KH176 will be more successful if the treated patients carry a missense mutation in a protein or in a protein subunit whose function directly depends on the availability of the CoQ cofactor. 

According to the above-reported comments, the limited clinical success of the cited molecules could be related to the administration of the investigated molecules to large and genetically heterogeneous cohorts of patients without taking into consideration the distance between the localization of the missense mutation responsible for the pathology and the expected site of action of the investigated CoQ analog. Furthermore, if the investigated molecules have to be administered to subjects affected by MDs for counteracting mitochondrial ROS production, the administration of the small molecules should be organelle-specific in order to reduce off-target effects, although some of those off-target effects may be apparently beneficial. 

To increase the delivery specificity of the cited molecules and cofactors to mitochondria, the same small molecules might be conjugated to triphenylphosphonium (TPP) salts [236] or can be entrapped in ad hoc functionalized liposomes [237]. Both systems should ensure the proper targeting of the small molecules to mitochondria, as already observed with the delivery of α-tocopherol or other small molecules, through conjugation to TPP or by embedding them in functionalized liposomes [236,237].

## 12. Conclusions

At the present, we are still far from developing an effective treatment for most of the MDs just mentioned, which share a severe neuromuscular and/or neurodegenerative phenotype and are characterized by a severe multisystem involvement still leading to a high mortality rate. Indeed, no real dedicated MD cure is available to date, although symptomatic therapies are established aiming to mitigate oxidative damage in tissues (i.e., brain, skeletal muscle, heart, liver, kidney) with primary mitochondrial dysfunction [49,63]. 

Within the vast group of inherited metabolic disorders, new strategies based on gene delivery and enzyme replacement approaches are under investigation [238,239], and therapeutic molecules that have been made available over the last few decades have significantly improved the prognosis quoad vitam and quoad valetudinem of affected patients [240].

Most of the employed symptomatic therapies are administered in combination with antioxidant cocktails. Conversely, according to what many authors have reported, the efficiency of antioxidant cocktails in patients affected by MDs has not been proven yet [36,37]. Furthermore, putative cytotoxic side-effects of therapies based on antioxidant administration need to be monitored for avoiding toxicity [36,37,78]. Indeed, the hypothesis that the suppression of endogenous oxidants could be deleterious and trigger local apoptosis/necrosis events cannot be excluded [36,37,78]. 

In order to evaluate and predict the possible advantages in administering specific cofactors, the most updated computational approaches based on molecular dynamics and energy stabilization calculations should be used [24,43,54,176,177,178,179,180,241,242,243,244,245,246,247], after having ascertained a real deficiency of these small molecules in the plasma and/or biopsy samples from patients. Indeed, while biopsy and serum blood levels of a specific cofactor can help in quantifying the real need for the antioxidant administration, computational approaches can help to understand if and how missense mutations affect the binding of a cofactor and, thus, to what extent the administered cofactor can successfully rescue the damage caused by a missense mutation in the investigated protein–cofactor binding area.

## Figures and Tables

**Table 1:** Hydrosoluble vitamin DRIs are reported according to https://www.ncbi.nlm.nih.gov/books/NBK222881/ and https://sinu.it/. An estimation of daily nutrient recommendations based on the DRI can be obtained by completing the linked form: https://www.nal.usda.gov/legacy/fnic/dri-calculator/index.php. Dietary supplement dosages for children and adults are coherent with those reported in https://ods.od.nih.gov/factsheets/PrimaryMitochondrialDisorders-HealthProfessional/. All the indicated web-links were accessed on 8 April 2022 for a final check.

Small Molecule	Vitamin and Coenzyme	DRI (mg/d)	Dosage for Children	Dosage for Adults	Food Sources (mg/serving; %Daily Value)	Web Reference
Thiamine	B1	0.4–1.3	10 mg/kg/day	100–1000 mg/day	Rice, white, long grain, enriched, parboiled, ½ cup (1.4; 117); Pork chop, bone-in, broiled, 3 ounces (0.4; 33); Trout, cooked, dry heat, 3 ounces (0.4; 33); Black beans, boiled, ½ cup (0.4; 33); Mussels, blue, cooked, moist heat, 3 ounces (0.3; 25); Tuna, Bluefin, cooked, dry heat, 3 ounces (0.2; 17); Macaroni, whole wheat, cooked, 1 cup (0.2; 17); Acorn squash, cubed, baked, ½ cup (0.2; 17); Rice, brown, long grain, not enriched, cooked, ½ cup (0.1; 8); Bread, whole wheat, 1 slice (0.1; 8); Orange juice, prepared from concentrate, 1 cup (0.1; 8); Sunflower seeds, toasted, 1 ounce (0.1; 8); Beef steak, bottom round, trimmed of fat, braised, 3 ounces (0.1; 8); Yogurt, plain, low fat, 1 cup (0.1; 8); Oatmeal, regular and quick, unenriched, cooked with water, ½ cup (0.1; 8); Corn, yellow, boiled, 1 medium ear (0.1; 8); Milk, 2%, 1 cup (0.1; 8); Barley, pearled, cooked, 1 cup (0.1; 8).	https://ods.od.nih.gov/factsheets/Thiamin-HealthProfessional/
Riboflavin	B2	50–400 mg po daily	50–400 mg po daily	50–400 mg/day (divided in 2–3 doses)	Beef liver, pan fried, 3 ounces (2.9; 223); Yogurt, plain, fat free, 1 cup (0.6; 46); Milk, 2% fat, 1 cup (0.5; 38); Beef, tenderloin steak, boneless, trimmed of fat, grilled, 3 ounces (0.4; 31); Clams, mixed species, cooked, moist heat, 3 ounces (0.4; 31); Mushrooms, portabella, sliced, grilled, ½ cup (0.3; 23); Almonds, dry roasted, 1 ounce (0.3; 23); Cheese, Swiss, 3 ounces (0.3; 23); Rotisserie chicken, breast meat only, 3 ounces (0.2; 15); Egg, whole, scrambled, 1 large (0.2; 15); Quinoa, cooked, 1 cup (0.2; 15); Salmon, pink, canned, 3 ounces (0.2; 15); Spinach, raw, 1 cup (0.1; 8); Apple, with skin, 1 large (0.1; 8); Kidney beans, canned, 1 cup (0.1; 8); Macaroni, elbow shaped, whole wheat, cooked, 1 cup (0.1; 8); Bread, whole wheat, 1 slice (0.1; 8); Cod, Atlantic, cooked, dry heat, 3 ounces (0.1; 8); Sunflower seeds, toasted, 1 ounce (0.1; 8); Tomatoes, crushed, canned, ½ cup (0.1; 8).	https://ods.od.nih.gov/factsheets/Riboflavin-HealthProfessional/
Niacin	B3 (PP)	5–14	25–250 mg/day	250 mg/day up to 1 g/day	Beef liver, pan fried, 3 ounces (14.9; 93); Chicken breast, meat only, grilled, 3 ounces (10.3; 64); Marinara (spaghetti) sauce, ready to serve, 1 cup (10.3; 64); Turkey breast, meat only, roasted, 3 ounces (10;0; 63); Salmon, sockeye, cooked, 3 ounces (8.6; 54); Tuna, light, canned in water, drained, 3 ounces (8.6; 54); Pork, tenderloin, roasted, 3 ounces (6.3; 39); Beef, ground, 90% lean, pan-browned, 3 ounces (5.8; 36); Rice, brown, cooked, 1 cup (5.2; 33); Peanuts, dry roasted, 1 ounce (4.2; 26); Potato (russet), baked, 1 medium (2.3; 14); Sunflower seeds, dry roasted, 1 ounce (2.0; 13); Bread, whole wheat, 1 slice (1.4; 9); Pumpkin seeds, dry roasted, 1 ounce (1.3; 8); Soymilk, unfortified, 1 cup (1.3; 8); Lentils, boiled and drained, ½ cup (1.0; 6); Bulgur, cooked, 1 cup (0.9; 6); Banana, 1 medium (0.8; 5); Edamame, frozen, prepared, ½ cup (0.7; 4); Raisins, ½ cup (0.6; 4); Tomatoes, cherry, ½ cup (0.5; 3); Broccoli, boiled, drained, chopped, ½ cup (0.4; 3); Cashews, dry roasted, 1 ounce (0.4; 3); Yogurt, plain, low fat, 1 cup (0.3; 2); Apple, 1 medium (0.2; 1); Chickpeas, canned, drained, 1 cup (0.2; 1); Milk, 1% milkfat, 1 cup (0.2; 1); Spinach, frozen, chopped, boiled, ½ cup (0.2; 1); Tofu, raw, firm, ½ cup (0.2; 1); Onions, chopped, ½ cup (0.1; 1).	https://ods.od.nih.gov/factsheets/Niacin-HealthProfessional/
Coenzyme Q10 (reduced, i.e., as ubiquinol, or oxidized, i.e., as ubiquinone)		3–6	CoQ_10_ as ubiquinol (preferred) 2–8 mg/kg po daily divided in two doses CoQ_10_ as ubiquinone 10–30 mg/kg po daily divided in 2 doses	CoQ_10_ as ubiquinol (preferred) 50–600 mg po daily CoQ_10_ as ubiquinone 300–2400 mg po daily divided 2–3 times a day	Sardines, salmon, trout, mackerel, 3 ounces (2.3–4/up to 50)). Chicken, beef, pork, 3 ounces (1.4–2.6/up to 50). Spinach, broccoli, cauliflower, ½ cup, chopped (0.4–0.5/up to 20). Fruits: strawberries, oranges, ½ cup (0.1–0.3/up to 20). Soybean and canola oils, 1 tablespoon (1.0–1.3/up to 30). Soybeans, lentils, peanuts, 1 ounce (0.6/up to 20). Pistachio, sesame seeds, 1 ounce (3.6, up to 80). Egg boiled, 1 medium (0.1/up to 3)	https://lpi.oregonstate.edu/mic/dietary-factors/coenzyme-Q10
Carnitine		NE	20–100 mg/kg/day divided into two or three doses	300–990 mg/dose two or three times per day	Beef steak, cooked, 4 ounces (56–162 mg); Ground beef, cooked, 4 ounces (87–99 mg); Milk, whole, 1 cup (8 mg); Codfish, cooked, 4 ounces (4–7 mg); Chicken breast, cooked, 4 ounces (3–5 mg); Ice cream, ½ cup (3 mg); Cheese, cheddar, 2 ounces (2 mg); Whole–wheat bread, 2 slices (0.2 mg); Asparagus, cooked, ½ cup (0.1 mg)	https://ods.od.nih.gov/factsheets/Carnitine-HealthProfessional/
Pantothenic acid	B5	2–7	B vitamin complexes 1 tab po given; one to two times per day	25 mg per day	Beef liver, boiled, 3 ounces (8.3; 166); Shitake mushrooms, cooked, ½ cup pieces (2.6; 52); Sunflower seeds, ¼ cup (2.4; 48); Chicken, breast meat, skinless, roasted, 3 ounces (1.3; 26); Tuna, fresh, bluefin, cooked, 3 ounces (1.2; 24); Avocados, raw, ½ avocado (1.0; 20); Milk, 2% milkfat, 1 cup (0.9; 18); Mushrooms, white, stir fried, ½ cup sliced (0.8; 16); Potatoes, russet, flesh and skin, baked, 1 medium (0.7; 14); Egg, hard boiled, 1 large (0.7; 14); Greek yogurt, vanilla, nonfat, 5.3-ounce container (0.6; 12); Ground beef, 85% lean meat, broiled, 3 ounces (0.6; 12); Peanuts, roasted in oil, ¼ cup (0.5; 10); Broccoli, boiled, ½ cup (0.5; 10); Whole-wheat pita, 1 large (0.5; 10); Chickpeas, canned, ½ cup (0.4; 8); Rice, brown, medium grain, cooked, ½ cup (0.4; 8); Oats, regular and quick, cooked with water, ½ cup (0.4; 8); Cheese, cheddar, 1.5 ounces (0.2; 4); Carrots, chopped, raw, ½ cup (0.2; 4); Cabbage, boiled, ½ cup (0.1; 2); Clementine, raw, 1 clementine (0.1; 2); Tomatoes, raw, chopped or sliced, ½ cup (0.1; 2).	https://ods.od.nih.gov/factsheets/PantothenicAcid-HealthProfessional/
Biotin	B7 (H)	5–30	5–10 mg/kg/ day	25 mg per day	Beef liver, cooked, 3 ounces (30.8; 103); Egg, whole, cooked (10.0; 33); Salmon, pink, canned in water, 3 ounces (5.0; 17); Pork chop, cooked, 3 ounces (3.8; 13); Hamburger patty, cooked, 3 ounces (3.8; 13); Sunflower seeds, roasted, ¼ cup (2.6; 9); Sweet potato, cooked, ½ cup (2.4; 8); Almonds, roasted, ¼ cup (1.5; 5); Tuna, canned in water, 3 ounces (0.6; 2); Spinach, boiled, ½ cup (0.5; 2); Broccoli, fresh, ½ cup (0.4; 1); Cheddar cheese, mild, 1 ounce (0.4; 1); Milk, 2%, 1 cup (0.3; 1); Plain yogurt, 1 cup (0.2; 1); Oatmeal, 1 cup (0.2; 1); Banana, ½ cup (0.2; 1).	https://ods.od.nih.gov/factsheets/Biotin-HealthProfessional/
Pyridoxine	B6	0.4–1.7	25 mg/day (generally in multivitamin complexes)	25 mg/day (generally in multivitamin complexes)	Chickpeas, canned, 1 cup (1.1; 65); Beef liver, pan fried, 3 ounces (0.9; 53); Tuna, yellowfin, fresh, cooked, 3 ounces (0.9; 53); Salmon, sockeye, cooked, 3 ounces (0.6; 35); Chicken breast, roasted, 3 ounces (0.5; 29); Potatoes, boiled, 1 cup (0.4; 25); Turkey, meat only, roasted, 3 ounces (0.4; 25); Banana, 1 medium (0.4; 25); Marinara (spaghetti) sauce, ready to serve, 1 cup (0.4; 25); Ground beef, patty, 85% lean, broiled, 3 ounces (0.3; 18); Waffles, plain, ready to heat, toasted, 1 waffle (0.3; 18); Bulgur, cooked, 1 cup (0.2; 12); Cottage cheese, 1% low-fat, 1 cup (0.2; 12); Squash, winter, baked, ½ cup (0.2; 12); Nuts, mixed, dry-roasted, 1 ounce (0.1; 6); Raisins, seedless, ½ cup (0.1; 6); Onions, chopped, ½ cup (0.1; 6); Spinach, frozen, chopped, boiled, ½ cup (0.1; 6); Tofu, raw, firm, prepared with calcium sulfate, ½ cup (0.1; 6); Watermelon, raw, 1 cup (0.1; 6).	https://ods.od.nih.gov/factsheets/VitaminB6-HealthProfessional/
Folic acid	B9	0.1–0.5	1 mg/day	1 mg/day	Beef liver, braised, 3 ounces (215 mcg; 54); Spinach, boiled, ½ cup (131 mcg; 33); Black-eyed peas (cowpeas), boiled, ½ cup (105 mcg; 26); Rice, white, medium-grain, cooked, ½ cup (90 mcg; 22); Asparagus, boiled, 4 spears (89 mcg; 22); Brussels sprouts, frozen, boiled, ½ cup (78 mcg; 20); Lettuce, romaine, shredded, 1 cup (64 mcg; 16); Avocado, raw, sliced, ½ cup (59 mcg; 15); Spinach, raw, 1 cup (58 mcg; 15); Broccoli, chopped, frozen, cooked, ½ cup (52 mcg; 13); Mustard greens, chopped, frozen, boiled, ½ cup (52 mcg; 13); Bread, white, 1 slice (50 mcg; 13); Green peas, frozen, boiled, ½ cup (47 mcg; 12); Kidney beans, canned, ½ cup (46 mcg; 12); Wheat germ, 2 tablespoons (40 mcg; 10); Tomato juice, canned, ¾ cup (36 mcg; 9); Crab, Dungeness, 3 ounces (36 mcg; 9); Orange juice, ¾ cup (35 mcg; 9); Turnip greens, frozen, boiled, ½ cup (32 mcg; 8); Peanuts, dry roasted, 1 ounce (27 mcg; 7); Orange, fresh, 1 small (29 mcg; 7) Papaya, raw, cubed, ½ cup (27 mcg; 7); Banana, 1 medium (24 mcg; 6); Yeast, baker’s, ¼ teaspoon (23 mcg; 6); Egg, whole, hard-boiled, 1 large (22 mcg; 6); Cantaloupe, raw, cubed, ½ cup (17 mcg; 4); Vegetarian baked beans, canned, ½ cup (15 mcg; 4); Fish, halibut, cooked, 3 ounces (12 mcg; 3); Milk, 1% fat, 1 cup (12 mcg; 3); Ground beef, 85% lean, cooked, 3 ounces (7 mcg; 2); Chicken breast, roasted, 3 ounces (3 mcg; 1).	https://ods.od.nih.gov/factsheets/Folate-HealthProfessional/
Cobalamine	B12	0.0007–0.0024	25 mcg/day (generally in multivitamin complexes)	25 mcg/day (generally in multivitamin complexes)	Beef liver, cooked, pan-fried, 3 ounces (70.7 mcg; 2944); Clams (without shells), cooked, 3 ounces (17 mcg; 708); Tuna, bluefin, cooked, dry heat, 3 ounces (9.3 mcg; 385); Salmon, Atlantic, cooked, 3 ounces (2.6 mcg; 108); Beef, ground, 85% lean meat/15% fat, pan-browned, 3 ounces (2.4 mcg; 100); Milk, 2% milkfat, 1 cup (1.3 mcg; 54); Yogurt, plain, fat free, 6-ounce container (1.0 mcg; 43); Cheese, cheddar, 1½ ounces (0.5 mcg; 19); Egg, whole, cooked, 1 large (0.5 mcg; 19); Turkey, breast meat, roasted, 3 ounces (0.3 mcg; 14); Tempeh, 1/2 cup (0.1 mcg; 3).	https://ods.od.nih.gov/factsheets/VitaminB12-HealthProfessional/
Ascorbic acid	C	25–90	5 mg/kg/day (or 10 mg qds)	50–200 mg po daily	Red pepper, sweet, raw, ½ cup (95; 106); Orange juice, ¾ cup (93; 103); Orange, 1 medium (70; 78); Grapefruit juice, ¾ cup (70; 78); Kiwifruit, 1 medium (64; 71); Green pepper, sweet, raw, ½ cup (60; 67); Broccoli, cooked, ½ cup (51; 57); Strawberries, fresh, sliced, ½ cup (49; 54); Brussels sprouts, cooked, ½ cup (48; 53); Grapefruit, ½ medium (39; 43); Broccoli, raw, ½ cup (39; 43); Tomato juice, ¾ cup (33; 37); Cantaloupe, ½ cup (29; 32); Cabbage, cooked, ½ cup (28; 31); Cauliflower, raw, ½ cup (26; 29); Potato, baked, 1 medium (17; 19); Tomato, raw, 1 medium (17; 19); Spinach, cooked, ½ cup (9; 10); Green peas, frozen, cooked, ½ cup (8; 9).	https://ods.od.nih.gov/factsheets/VitaminC-HealthProfessional/

**Table 2:** Liposoluble (hydrophobic) vitamins: DRIs are reported according to https://www.ncbi.nlm.nih.gov/books/NBK222881/ and https://sinu.it/. An estimation of daily nutrient recommendations based on the DRI can be obtained by completing the linked form: https://www.nal.usda.gov/legacy/fnic/dri-calculator/index.php; https://ods.od.nih.gov/factsheets/VitaminE-HealthProfessional/. Dietary supplement dosages for children and adults are coherent with those reported in the cited references within paragraphs dedicated to each vitamin (see below) and/or with those reported in https://ods.od.nih.gov/factsheets/PrimaryMitochondrialDisorders-HealthProfessional/. All the indicated web-links were accessed on 8 April 2022 for a final check.

Small Molecule	Vitamin and Coenzyme	DRI (mg/d)	Dosage for Children	Dosage for Adults	Food Sources (mcg/serving; % Daily Value)	Web Reference
Lipoic acid		50–600	50–200 mg/day	50–200 mg/day	Beef kidney, heart and liver (1–3 mcg/g dry wt) as lipoyllysine); Spinach and broccoli (1–3 mcg/g dry wt, as lipoyllysine); Tomatoes, peas and brussels sprouts (0.5 mcg/g dry wt, as lip).	https://lpi.oregonstate.edu/mic/dietary-factors/lipoic-acid
Retinol	A	0.7–1.3	0.3–0.9 mg per day (depending on the age)	Up to 3 mg per day	Beef liver, pan fried, 3 ounces (6582 mcg; 731); Sweet potato, baked in skin, 1 whole (1403 mcg; 156); Spinach, frozen, boiled, ½ cup (573 mcg; 64); Pumpkin pie, commercially prepared, 1 piece (488 mcg; 54); Carrots, raw, ½ cup (459 mcg; 51); Ice cream, French vanilla, soft serve, 1 cup (278 mcg; 31); Cheese, ricotta, part skim, 1 cup (263 mcg; 29); Herring, Atlantic, pickled, 3 ounces (219 mcg; 24); Cantaloupe, raw, ½ cup (135 mcg; 15); Peppers, sweet, red, raw, ½ cup (117 mcg; 13); Mangos, raw, 1 whole (112 mcg; 12); Egg, hard boiled, 1 large (75 mcg; 8); Black-eyed peas (cowpeas), boiled, 1 cup (66 mcg; 7); Apricots, dried, sulfured, 5 apricots (63 mcg; 7); Broccoli, boiled, ½ cup (60 mcg; 7); Salmon, sockeye, cooked, 3 ounces (59 mcg; 7); Tomato juice, canned, ¾ cup (42 mcg; 5); Yogurt, plain, low fat, 1 cup (32 mcg; 4); Tuna, light, canned in oil, drained solids, 3 ounces (20 mcg; 2); Baked beans, canned, plain or vegetarian, 1 cup (13 mcg; 1); Summer squash, all varieties, boiled, ½ cup (10 mcg; 1); Chicken, breast meat and skin, roasted, ½ breast (5 mcg; 1); Pistachio nuts, dry roasted, 1 ounce (4 mcg; 0).	https://ods.od.nih.gov/factsheets/VitaminA-HealthProfessional/
Calciferol	D	0.1–0.2	0.25–1 mg	1 mg	Cod liver oil, 1 tablespoon (34.0; 170); Trout (rainbow), farmed, cooked, 3 ounces (16.2 mcg; 81); Salmon (sockeye), cooked, 3 ounces (14.2 mcg; 71); Mushrooms, white, raw, sliced, exposed to UV light, ½ cup (9.2 mcg; 46); Sardines (Atlantic), canned in oil, drained, 2 sardines (1.2 mcg; 6); Egg, 1 large, scrambled (1.1 mcg; 6); Liver, beef, braised, 3 ounces (1.0 mcg; 5); Tuna fish (light), canned in water, drained, 3 ounces (1.0 mcg; 5); Cheese, cheddar, 1.5 ounce (0.4 mcg; 2); Mushrooms, portabella, raw, diced, ½ cup (0.1 mcg; 1); Chicken breast, roasted, 3 ounces (0.1 mcg; 1).	https://ods.od.nih.gov/factsheets/VitaminD-HealthProfessional/
α-Tocopherol	E	4–19	1–2 IU/kg po daily	100–200 IU po daily	Wheat germ oil, 1 tablespoon (20.3; 135); Sunflower seeds, dry roasted, 1 ounce (7.4; 49); Almonds, dry roasted, 1 ounce (6.8; 45); Sunflower oil, 1 tablespoon (5.6; 37); Safflower oil, 1 tablespoon (4.6; 31); Hazelnuts, dry roasted, 1 ounce (4.3; 29); Peanut butter, 2 tablespoons (2.9;19); Peanuts, dry roasted, 1 ounce (2.2; 15); Corn oil, 1 tablespoon (1.9; 13); Spinach, boiled, ½ cup (1.9; 13); Broccoli, chopped, boiled, ½ cup (1.2; 8); Soybean oil, 1 tablespoon (1.1; 7); Kiwifruit, 1 medium (1.1; 7); Mango, sliced, ½ cup (0.7; 5); Tomato, raw, 1 medium (0.7; 5); Spinach, raw, 1 cup (0.6; 4).	https://ods.od.nih.gov/factsheets/VitaminE-HealthProfessional/
Phylloquinone	K	0.09–0.12	2–75 mcg	90–120 mcg	Natto, 3 ounces (as MK-7; 850 mcg; 708); Collards, frozen, boiled, ½ cup (530 mcg; 442); Turnip greens, frozen, boiled ½ cup (426 mcg; 355); Spinach, raw, 1 cup (145 mcg; 121); Kale, raw, 1 cup (113 mcg; 94); Broccoli, chopped, boiled, ½ cup (110 mcg; 92); Soybeans, roasted, ½ cup (43 mcg; 36); Carrot juice, ¾ cup (28 mcg; 23); Soybean oil, 1 tablespoon (25 mcg; 21); Edamame, frozen, prepared, ½ cup (21 mcg; 18); Pumpkin, canned, ½ cup (20 mcg; 17); Pomegranate juice, ¾ cup (19 mcg; 16); Okra, raw, ½ cup (16 mcg; 13); Salad dressing, Caesar, 1 tablespoon (15 mcg; 13); Pine nuts, dried, 1 ounce (15 mcg; 13); Blueberries, raw, ½ cup (14 mcg; 12); Iceberg lettuce, raw, 1 cup (14 mcg; 12); Chicken, breast, rotisserie, 3 ounces (as MK-4; 13 mcg; 11); Grapes, ½ cup (11 mcg; 9); Vegetable juice cocktail, ¾ cup (10 mcg; 8); Canola oil, 1 tablespoon cup (10 mcg; 8); Cashews, dry roasted, 1 ounce cup (10 mcg; 8); Carrots, raw, 1 medium (8 mcg; 7); Olive oil, 1 tablespoon (8 mcg; 7); Ground beef, broiled, 3 ounces (as MK-4; 6 mcg; 5); Figs, dried, ¼ cup (6 mcg; 5); Chicken liver, braised, 3 ounces (as MK-4; 6 mcg; 5); Ham, roasted or pan-broiled, 3 ounces (as MK-4; 4 mcg; 3); Cheddar cheese, 1½ ounces (as MK-4; 4 mcg; 3); Mixed nuts, dry roasted, 1 ounce (4 mcg; 3); Egg, hard boiled, 1 large (as MK-4; 4 mcg; 3); Mozzarella cheese, 1½ ounces (as MK-4; 2 mcg; 2); Milk, 2%, 1 cup (as MK-4; 1 mcg; 1); Salmon, sockeye, cooked, 3 ounces (as MK-4; 0.3 mcg; 0); Shrimp, cooked, 3 ounces (as MK-4; 0.3 mcg; 0).	https://ods.od.nih.gov/factsheets/vitaminK-HealthProfessional/

**Table 3:** List of the main mitochondrial/cytosolic proteins involved in interactions with the reported hydrophilic vitamin-related cofactors. All the indicated web-links were accessed on 8 April 2022 for a final check.

Small Molecule	Related Cofactor	Cofactor Protein Associations	Gene Name	Protein Targets from HMDB
Thiamine—B1	Thiamine monophosphate	Cancer-related nucleoside-triphosphatase; membrane	*NTPCR*	https://hmdb.ca/metabolites/HMDB0002666/metabolite_protein_links
		14 kDa phosphohistidine phosphatase; cytosol	*PHPT1*	
	Thiamine pyrophosphate	Pyruvate dehydrogenase E1	*PDH*	https://hmdb.ca/metabolites/HMDB0001372/metabolite_protein_links
		2-Oxoisovalerate dehydrogenase	*BCKDH*	
		2-Oxoglutarate dehydrogenase	*OGDH*	
		2-Oxoglutarate dehydrogenase-like	*OGDHL*	
		Thiamine pyrophosphate carrier	*SLC25A19*	
Riboflavin—B2	FMN	Dihydroorotate dehydrogenase	*DHODH*	https://hmdb.ca/metabolites/HMDB0001520/metabolite_protein_links
		NADH dehydrogenase UQ flavoprotein 1	*NDUFV1*	
		Sarcosine dehydrogenase	*SARDH*	
	FAD	FAD (and/or folate) carrier	*SLC25A32*	https://hmdb.ca/metabolites/HMDB0001248/metabolite_protein_links
		Dihydrolipoyl dehydrogenase	*DLD*	
		Acyl carrier protein	*NDUFAB1*	
		Long-chain specific acyl-CoA dehydrogenase	*ACADL*	
		Short-chain specific acyl-CoA dehydrogenase	*ACADS*	
		Medium-chain specific acyl-CoA dehydrogenase	*ACADM*	
		NADH-ubiquinone oxidoreductase	*MT-ND*	
		Succinate dehydrogenase	*SDHD*	
		Electron-transport flavoprotein dehydrogenase	*IVD*	
		Thioredoxin reductase 2	*ETFDH*	
		Isovaleryl-CoA dehydrogenase	*TXNRD2*	
		Dihydroorotate dehydrogenase	*DHODH*	
		Glutathione reductase	*GSR*	
		Glycerol-3-phosphate dehydrogenase	*GPD2*	
		Choline dehydrogenase	*CHDH*	
		Glutaryl-CoA dehydrogenase	*GCDH*	
		Dimethylglycine dehydrogenase,	*DMGDH*	
		Sarcosine dehydrogenase	*SARDH*	
		Proline dehydrogenase 1	*PRODH*	
		Hydroxyglutarate dehydrogenase	*HGDH*	
		Apoptosis-inducing factor 1	*AIFM1*	
		Probable D-lactate dehydrogenase	*LDHD*	
		Isobutyryl-CoA dehydrogenase	*ACAD8*	
		Protein MTO1 homolog	*MTO1*	
		(Pyruvate dehydrogenase (acetyl-transferring))-phosphatase 2	*PDP2*	
		Sulfide:quinone oxidoreductase	*SQRDL*	
Niacin—B3	NMN	5′(3′)-deoxyribonucleotidase	*NT5M*	https://hmdb.ca/metabolites/HMDB0000229/metabolite_protein_links
	NADH	Pyruvate dehydrogenase	*PDH*	https://hmdb.ca/metabolites/HMDB0000902/metabolite_protein_links
		Methylmalonate-semialdehyde dehydrogenase	*ALDH6A1*	
		Dihydrolipoyl dehydrogenase	*DLD*	
		Acyl-CoA dehydrogenase	*ACAD*	
		NADH-ubiquinone oxidoreductase	*MT-ND*	
		Delta-1-pyrroline-5-carboxylate dehydrogenase	*ALDH4A1*	
		Acyl carrier protein	*NDUFAB1*	
		Glutathione reductase	*GSR*	
		Succinate-semialdehyde dehydrogenase	*ALDH5A1*	
		Aldehyde dehydrogenase	*ALDH2*	
		Pyrroline-5-carboxylate reductase 1	*PYCR1*	
		Trifunctional enzyme	*HADH*	
		3-Hydroxyisobutyrate dehydrogenase	*HIBADH*	
		2-Oxoglutarate dehydrogenase	*OGDH*	
		D-beta-hydroxybutyrate dehydrogenase	*BDH1*	
		Glutamate dehydrogenase	*GLUD*	
		Aminomethyltransferase	*AMT*	
		Alpha-aminoadipic semialdehyde synthase	*AASS*	
		Malate dehydrogenase	*MDH*	
		NAD-dependent malic enzyme	*ME*	
		Isocitrate dehydrogenase	*IDH3*	
		NAD(P) transhydrogenase	*NNT*	
		Bifunctional methylenetetrahydrofolate dehydrogenase/cyclohydrolase	*MTHFD2*	
		Tricarboxylate transport protein	*SLC25A1*	
		Proline dehydrogenase 1	*PRODH*	
		Glycine cleavage system H protein	*GCSH*	
		NAD-dependent protein deacetylase sirtuin-3	*SIRT3*	
		NAD-dependent protein deacetylase sirtuin-5	*SIRT5*	
		2-Oxoglutarate dehydrogenase-like	*OGDHL*	
		Dihydrofolate reductase	*DHFRL1*	
		NAD kinase domain-containing protein 1	*NADKD1*	
Ubiquinone/coenzyme Q_10_ (reduced, i.e., as ubiquinol, or oxidized, i.e., as ubiquinone)	UQ/CoQ_10_ (reduced, i.e., UQH_2_)	NADH dehydrogenase (ubiquinone)	*NDFU*	https://hmdb.ca/metabolites/HMDB0001072/metabolite_protein_links and https://hmdb.ca/metabolites/HMDB0001304/metabolite_protein_links
		NADH-ubiquinone oxidoreductase	*MT-ND*	
		Succinate dehydrogenase	*SDH*	
		Acyl carrier protein	*NDUFA5*	
		Cytochrome b-c1 complex	*UQCR*	
		Electron transfer flavoprotein-ubiquinone oxidoreductase	*ETFDH*	
		Cytochrome c1, heme protein	*CYC1*	
Carnitine, acetyl-L-carnitine		Carnitine O-acetyltransferase	*CRAT*	https://hmdb.ca/metabolites/HMDB0000201/metabolite_protein_links and https://hmdb.ca/metabolites/HMDB0000062/metabolite_protein_links
		Carnitine O-palmitoyltransferase 2	*CPT2*	
		Carnitine/acylcarnitine carrier	*SLC25A20*	
Pantothenic acid—B5		Pantothenate kinase 2	*PANK2*	https://hmdb.ca/metabolites/HMDB0000210/metabolite_protein_links
Pyridoxine—B6		Alpha-aminoadipic semialdehyde dehydrogenase	*ALDH7A1*	https://hmdb.ca/metabolites/HMDB0000239/metabolite_protein_links
Biotin—B7		Pyruvate carboxylase	*PC*	https://hmdb.ca/metabolites/HMDB0000030/metabolite_protein_links
		Propionyl-CoA carboxylase	*PCC*	
		Methylcrotonoyl-CoA carboxylase	*MCCC*	
Folic acid—B9		Folate (and/or FAD) carrier	*SLC25A32*	https://hmdb.ca/metabolites/HMDB0000121/metabolite_protein_links
		Dihydrofolate reductase	*DHFRL1*	
Cobalamine—B12		Methylmalonyl-CoA mutase	*MUT*	https://hmdb.ca/metabolites/HMDB0002174/metabolite_protein_links
		Methylmalonic aciduria type A protein	*MMAA*	
Ascorbic acid—C		Trimethyllysine dioxygenase	*TMLHE*	https://hmdb.ca/metabolites/HMDB0000044/metabolite_protein_links

**Table 4:** List of the main mitochondrial/cytosolic proteins involved in interactions with the reported lipophilic vitamin-related cofactors. All the indicated web-links were accessed on 8 April 2022 for a final check.

Small Molecule/Cofactor	Cofactor Protein Associations	Gene Name	Protein Targets from HMDB
Lipoic acid	Lipoyl synthase	*LIAS*	https://hmdb.ca/metabolites/HMDB0014312/metabolite_protein_links
	Acyl-coenzyme A synthetase ACSM1	*ACSM1*	
	Lipoyltransferase 1	*LIPT1*	
Retinol—A	Diacylglycerol O-acyltransferase 1; endoplasmic reticulum	*DGAT1*	https://hmdb.ca/metabolites/HMDB0000305/metabolite_protein_links
	Retinol dehydrogenase; endoplasmic reticulum	*RDH*	
	Alcohol dehydrogenase; cytoplasm	*ADH*	
	Lecithin retinol acyltransferase; endoplasmic reticulum and other locations	*LRAT*	
	17-Beta-hydroxysteroid dehydrogenase type 6; endosome and endoplasmic reticulum	*HSD17B6*	
	Acyl-CoA wax alcohol acyltransferase; endoplasmic reticulum	*AWAT*	
	All-trans-retinol 13,14-reductase; endoplasmic reticulum	*RETSAT*	
	Short-chain dehydrogenase/reductase 3; membrane	*DHRS3*	
	Epidermal retinol dehydrogenase 2; endoplasmic reticulum	*SDR16C5*	
Calciferol—D	Cholesterol side-chain cleavage enzyme	*CYP11A1*	https://hmdb.ca/metabolites/HMDB0000876/metabolite_protein_links
	Sterol 26-hydroxylase	*CYP27A1*	
	25-Hydroxyvitamin D-1 alpha hydroxylase	*CYP27B1*	
	1,25-Dihydroxyvitamin D(3) 24-hydroxylase	*CYP24A1*	
	UMP-CMP kinase 2	*CMPK2*	
α-Tocopherol—E	Superoxide dismutase (Cu-Zn)	*SOD1*	https://hmdb.ca/metabolites/HMDB0001893/metabolite_protein_links
Phylloquinone—K	NAD(P)H dehydrogenase (quinone) 1; cytoplasm	*NQO1*	https://hmdb.ca/metabolites/HMDB0003555/metabolite_protein_links
	Vitamin K-dependent gamma-carboxylase; endoplasmic reticulum	*GCCX*	
	Osteocalcin; secreted	*BGLAP*	
	Vitamin K epoxide reductase complex subunit 1; endoplasmic reticulum	*VKORC1*	

**Table 5:** Gene variants analyzed in our laboratories associated with the reported syndromes. Mutation nucleotide-numbering is based on reference sequences (RefSeq) for nuclear genes and on the genomic Revised Cambridge Reference Sequence (rCRS) sequence (RefSeq accession number NC_012920.1) for mitochondrial genes. “*” indicates a premature protein truncation.

Gene	NT Mutation	AA Mutation	Syndromes
*MT-ND5*	m.13513G>A	D393N	LS, MELAS/LS overlap syndrome
*MT-ND5*	m.13042G>A	A236T	LS, MERRF/MELAS syndrome, LHON-like syndrome
*NDUFAF6*	compound heterozygosity, NM_152416.4: c.532G>C; p.A178P; deep intronic c.420 + 784C>T NM_152416.4: c.371T>C; p.I124T	A178P + intron I124T	LS
*MT-TK*	m.8344A>G	na	MERRF, LS
*MT-TK*	m.8363G>A	na	MERRF, Cardiomiopathy and deafness, LS
*MT-TL1*	m.3243A>G	na	MELAS, MIDD, PEO
*MT-TF*	m.641A>T	na	Mitochondrial encephalomyopathy
*CRAT*	compound heterozygosity, NM_000755.4: c.329A>G, p.Y110C c.1705G>A, p.V569M	Y110C; V569M	Mitochondrial encephalomyopathy
*SLC25A10*	compound heterozygosity, NM_001270888.1: c.304AZT, p.K102 *; c.684C>T, p.P228P; intronic c.790-37G>A	K102 *; P228P + intronic	Mitochondrial encephalomyopathy
*EARS2*	compound heterozygosity, NM_001083614.2 c.322C>T, p.R108W; c.502A>G, p.R168G; hexon 7 insertion c.1278_1279insCTC, p.Leu427ins	R108W; R168G + L427ins	Leukoencephalopathy with thalamus and brainstem involvement and high lactate (LTBL)
*SERAC1*	Homozygous, NM_03286.1 c.1709 G>A; p.G526E; c. 1643_1646 dup ATCT, p.Leu550-SerfsX19; c.1593T>G, p.S531R	G526E L550-SfsX19; S531R	MEGDEL

**Table 6:** List of the aminoacyl tRNA synthetases crystallized in complex with a transported amino acid.

PDB_ID	Aminoacyl tRNA Synthetase Name	Number of Residues	Aminoacyl tRNA	Number of Nucleotides	Organism	Cell Compartment	Resolution
1f7u (1f7v)	Arginyl-tRNA synthetase	608 (607)	Arg tRNA	76	*S. cerevisiae*	cytoplasmic/mitochondrion	2.20 Å (2.90 Å)
2zue (2zuf)	Arginyl-tRNA synthetase	629	Arg tRNA	78	*P. horikoshii*	cytoplasmic	2.00 Å (2.30 Å)
3kfu	Glutamyl-tRNA amidotransferase subunit A, aspartyl/Glutamyl-tRNA amidotrasferase subunit B, Glutamyl amidotrasferase subunit C-tRNA	471, 466, 92	Asn tRNA	76	*T. thermophilus HB8*	cytoplasmic	3.00 Å
1c0a	Aspartyl-tRNA synthetase	585	Asp tRNA	77	*E. coli*	cytoplasmic	2.40 Å
1efw	Aspartyl-tRNA synthetase	580	Asp tRNA	73	*T. thermpohilus/E. coli*	cytoplasmic	3.00 Å
1il2	Aspartyl-tRNA synthetase	590	Asp tRNA	75	*E. coli/S. cerevisiae*	cytoplasmic/mitochondrion	2.60 Å
1u0b	Cysteinyl-tRNA synthetase	461	Cys tRNA	74	*E. coli*	cytoplasmic	2.30 Å
1exd	Glutaminyl-tRNA synthetase	548	Gln Aptamer tRNA	73	*E. coli*	cytoplasmic	2.70 Å
3aL0	Glutamyl-tRNA amidotransferase subunit A	475	Gln tRNA	74	*T. maritima MSB8*	cytoplasmic	3.37 Å
1euy (1euq)	Glutaminyl-tRNA synthetase	548	Gln tRNA	74 (72)	*E. coli*	cytoplasmic	2.60 Å (3.10 Å)
1gsg	Glutaminyl-tRNA synthetase	553	Gln tRNA	75	*E. coli*	cytoplasmic	2.80 Å
1gts	Glutaminyl-tRNA (E.C.6.1.1.18) synthetase	553	Gln tRNA	74	*E. coli*	cytoplasmic	2.80 Å
1o0b (1o0c)	Glutaminyl-tRNA synthetase	554	Gln tRNA	75	*E. coli*	cytoplasmic	2.70 Å (2.70 Å)
1zjw	Glutaminyl-tRNA synthetase	553	Gln tRNA	75	*E. coli*	cytoplasmic	2.50 Å
2rd2	Glutaminyl-tRNA synthetase	556	Gln tRNA	75	*E. coli K-12 (E. coli)*	cytoplasmic	2.60 Å
3akz	Glutamyl-tRNA synthetase2	487	Gln tRNA		*T. maritima MSB8*	cytoplasmic	2.90 Å
1qrs (1qrt, 1qru)	Glutaminyl-tRNA (E.C.6.1.1.18) synthetase	553	Gln2 tRNA	75	*E. coli*	cytoplasmic	2.60 Å (2.70 Å, 3.00 Å
1qtq	Glutaminyl-tRNA synthetase	553	Gln2 tRNA	75	*E. coli*	cytoplasmic	2.25 Å
1g59	Glutamyl-tRNA synthetase	648	Glu tRNA	75	*T. thermophilus*	cytoplasmic	2.40 Å
1n78 (1n77, 1j09, 1n78)	Glutamyl-tRNA synthetase	468	Glu tRNA (Glu tRNA, /, /)	75 (75, /, /)	*T. thermophilus*	cytoplasmic	2.10 Å (2.40 Å, 1.80 Å, 1.90 Å)
4kr2 (4kr3)	Glycine-tRNA synthetase	637	Gly tRNA-ccc	74	*H. sapiens*	cytoplasmic/mitochondria	3.29 Å (3.31 Å)
5e6m	Glycine-tRNA synthetase	693	Gly tRNA-ccc	74	*H. sapiens*	cytoplasmic/mitochondria	2.93 Å
4qei (4kqe)	Glycine-tRNA synthetase	630	Gly tRNA-ccc-2-2 (/)	69 (/)	*H. sapiens*	cytoplasmic/mitochondria	2.88 Å
4rdx	Histidine-tRNA synthetase	423	His tRNA	78	*T. thermophilus HB27*	cytoplasmic	2.55 Å
1ffy (1qu2, 1qu3)	Isoleucyl-tRNA synthetase	917	Ile tRNA	75	*S. aureus*	cytoplasmic	2.20 Å (2.20 Å, 2.90 Å)
1wz2	Leucyl-tRNA synthetase	967	Leu tRNA	88	*P. horikoshii OT3*	cytoplasmic	3.21 Å
3zjt (3zjv, 3zju)	Leucyl-tRNA synthetase	880	Leu5 tRNA UAA isoacceptor	88 (87, 87)	*E. coli/E. coli K-12*	cytoplasmic	2.20 Å (2.30 Å, 2.40 Å)
4arc (4asi, 4ari, 4aq7)	Leucyl-tRNA synthetase	880	Leu5 tRNA UAA isoacceptor	87	*E. coli K-12*	cytoplasmic/mitochondria	2.00 Å (2.02 Å, 2.08 Å, 2.50 Å)
5ah5	Leucyl-tRNA synthetase	822	Leu tRNA UAA isoacceptor	84	*A. radiobacter K84*	cytoplasmic	2.10 Å
3zgz	Leucyl-tRNA synthetase	880	Leu5 tRNA UAA isoacceptor	88	*E. coli* *K-12*	cytoplasmic	2.40 Å
4cqn	Leucyl-tRNA synthetase	880	Leu5 tRNA UAA isoacceptor	82	*E. coli K-12*	cytoplasmic	2.50 Å
7k98 (7kao, 7k9m, 7kab)	Phenylalanyl-tRNA synthetase	344	Phe tRNA	77	*M. tubercolosis H37Rv*	cytoplasmic	2.19 Å (2.40 Å, 2.50 Å, 2.50 Å)
3tup	Phenylalanyl-tRNA synthetase	415	Phe tRNA	76	*H. sapiens*	cytoplasmic/mitochondria	3.05 Å
1eiy	Phenylalanyl-tRNA synthetase	350	Phe tRNA	76	*T. thermophilus HB8*	cytoplasmic	3.30 Å
3w3s	Seryl-tRNA type-2 synthetase	527	Selenocysteine tRNA	99	*M. kandleri AV19*	cytoplasmic	3.10 Å
4rqf (4rqe)	Seryl-tRNA synthetase	522	Selenocysteine tRNA	90	*H. sapiens*	cytoplasmic	3.50 Å (4.00 Å)
1ser	Seryl-tRNA synthetase E.C.6.1.1.11	421	Ser tRNA	94	*T. thermophilus*	cytoplasmic	2.90 Å
1qf6	Threonyl-tRNA synthetase	642	Thr tRNA	76	*E. coli*	cytoplasmic	2.90 Å
1kog	Threonyl-tRNA synthetase	401	Thr tRNA synthetase mRNA	37	*E. coli*	cytoplasmic	3.50 Å
2ake (2dr2)	Tryptophanyl-tRNA synthetase	384	Trp transfer tRNA	72 (75)	*H. sapiens/B. taurus*	cytoplasmic	3.10 Å (2.40)
1j1u	Tyrosyl-tRNA synthetase	306	Tyr tRNA	77	*M. jannaschii*	cytoplasmic	1.95 Å
1ivs (1iyw)	Valyl-tRNA synthetase	862	Val tRNA (/)	75 (/)	*T. thermophilus*	cytoplasmic	2.90 Å (4.00 Å)
1gax	Valyl-tRNA synthetase	862	Val tRNA	75	*T. thermophilus*	cytoplasmic	2.90 Å
2cv2	Glutamyl-tRNA synthetase	468	tRNA	75	*T. thermophilus*	cytoplasmic	2.69 Å
2dxi (2cv0, 2cv2, 2cuz)	Glutamyl-tRNA synthetase	468	tRNA (tRNA, tRNA, /)	75 (75, 75, /)	*T. thermophilus*	cytoplasmic	2.20 Å (2.40 Å, 2.40 Å, 1.98 Å)
2d6f	Glutamyl-tRNA(Gln) amidotransferase subunit D	435	tRNA	74	*M. thermautrophicus*	cytoplasmic	3.15Å
2du3 (2du4, 2du5, 2du6)	O-Phosphoseryl-tRNA synthetase	534	tRNA	71	*A. fulgidus*	/	2.60 Å (2.80Å, 3.20 Å, 3.20 Å)
2du7	O-Phosphoseryl-tRNA synthetase	549	/	/	*M. jannaschii*	cytoplasmic	3.60 Å
4yye	Threonyl-tRNA synthetase	460	tRNA	76	*S. cerevisiae S288c*	cytoplasmic/mitochondria	2.30 Å
5yyn	Arginyl-tRNA synthetase	586	tRNA	77	*E. coli K-12*	cytoplasmic	3.00Å
2zni	Pyrrolysyl-tRNA synthetase	308	Bacterial tRNA	72	*D. hefniense*	cytoplasmic	3.10 Å
3a2k	Lysidine-tRNA synthase	464	Bacterial tRNA		*G. kaustophilus*	cytoplasmic	3.65 Å
2hrk (2hsn, 2hsm)	Glutamyl-tRNA synthetase	207	/	/	*S. cerevisiae*	cytoplasmic/mitochondria	2.05 Å (2.20 Å, 3.00 Å)
2rkj	Tyrosyl-tRNA synthetase	392	238-MER RNA	246	*N. crassa*	cytoplasmic/mitochondria	4.50 Å
5ud5 (5v6x)	Pyrrolysyl-tRNA synthetase	109	70-MER RNA	72	*M.* *mazei Go1*	cytoplasmic	2.35 Å (2.76 Å)
4jxx (4jxz, 4jyzx)	Glutaminyl-tRNA synthetase	553	71-MER RNA (71-MER RNA, 72-MER RNA)	75	*E.* *coli K-12*	cytoplasmic	2.30 Å (2.40Å, 2.50 Å)
2azx	Tryptophanyl-tRNA synthetase	477	72-MER	75	*H. sapiens*	cytoplasmic	2.80 Å
2csx (2ct8)	Methionyl-tRNA synthetase	497	75-MER RNA (74-MER RNA)	75 (74)	*A. aeolicus*	cytoplasmic	2.70 Å (2.70 Å)
3wqy (3wqz)	Alenine-tRNA synthetase	906	75-MER RNA	75	*A. filgidus DSM 4304*	cytoplasmic	3.30 Å (3.49 Å)
4wj3 (4wj4)	Glutamyl-tRNA(Gln) amidotransferase subunit A	484 (599)	76-MER tRNA	76	*P.* *aeruginosa* *PAO1*	cytoplasmic	3.71 Å (3.29 Å)

## Data Availability

Not applicable.

## Abbreviations

AAC, ADP/ATP carrier; ACP, acyl-carrier protein; AGC, aspartate/glutamate carrier; AIF, apoptosis-inducing factor; Bak, Bcl-2 antagonist/killer-1; Bax, Bcl-2 associated X protein; Bcl-2, B-cell lymphoma-2; BDC, biotin-dependent carboxylases; BTBGD, biotin-thiamine-responsive basal ganglia disease; CAC, carnitine/acyl-carnitine carrier; Cbl, Cobalamin; CFD, cerebral folate deficiency; CI, complex I; CIC, citrate carrier; CII, complex II; CIII, complex III; CIV, complex IV; CV, complex V; CoA, coenzyme A; CoQ, ubiquinone; CoQ_10_, coenzyme Q_10_; CoQH_2_, ubiquinol; CPEO, chronic progressive external ophthalmoplegia; CRAT, carnitine acetyltransferase; CypD, cyclophilin D; CytC, cytochrome C; DIC, dicarboxylate carrier; DRI, dietary reference intake; ETC, electron transport chain; ETF, electron transfer flavoprotein; ETFDH, electron-transport flavoprotein dehydrogenase; FAD, flavin adenine dinucleotide; FMN, flavin mononucleotide; GLUT1, glucose transporter 1; IMS, intermembrane space; KSS, Kearns–Sayre syndrome; LHON, Leber’s hereditary optic neuropathy; MADD, multiple acyl CoA dehydrogenase deficiency; MAS, malate/aspartate shuttle; MEGDEL syndrome, 3-methylglutaconic acidemia, deafness, encephalopathy and Leigh-like syndrome; MELAS, stroke-like episodes; MERRF, myoclonic epilepsy with ragged-red fibers; 5-MTHF, 5-methyl-THF; MDs, mitochondrial diseases; MFT, FAD (folate/riboflavin/FAD) carrier; MIM, mitochondrial inner membrane; MOM, mitochondrial outer membrane; MPC, mitochondrial pyruvate carrier; MPC, mitochondrial pyruvate carrier; NAD+, nicotinamide adenine dinucleotide; NADP^+^, nicotinamide adenine dinucleotide phosphate; NARP, neuropathy, ataxia and retinitis pigmentosa; NDT, NAD^+^ carrier; NMN, nicotinamide mononucleotide; OGC, malate/2-oxoglutarate carrier; ORC, ornithine carrier; OXPHOS, mitochondrial oxidative phosphorylation; PANK, pantothenate kinase; PiC, phosphate carrier; PLP, pyridoxal phosphate; PNC, pyrimidine nucleotide carrier; RDA, recommended dietary allowance; ROS, reactive oxygen species; SERAC1, serine active site-containing protein 1; TCA, tricarboxylic acid cycle; THF, tetrahydrofolate; TPC, thiamine pyrophosphate carrier; TPP, triphenylphosphonium; UCP, uncoupling protein; VDAC, voltage-dependent anion channel.
